# Biased Retention of Environment-Responsive Genes Following Genome Fractionation

**DOI:** 10.1093/molbev/msae155

**Published:** 2024-07-29

**Authors:** Marc Beringer, Rimjhim Roy Choudhury, Terezie Mandáková, Sandra Grünig, Manuel Poretti, Ilia J Leitch, Martin A Lysak, Christian Parisod

**Affiliations:** Department of Biology, University of Fribourg, Chemin du Musée 10, 1700 Fribourg, Switzerland; Institute of Plant Sciences, University of Bern, Altenbergrain 21, 3013 Bern, Switzerland; Department of Biology, University of Fribourg, Chemin du Musée 10, 1700 Fribourg, Switzerland; Institute of Plant Sciences, University of Bern, Altenbergrain 21, 3013 Bern, Switzerland; Central European Institute of Technology, Masaryk University, 625 00 Brno, Czech Republic; Department of Biology, University of Fribourg, Chemin du Musée 10, 1700 Fribourg, Switzerland; Institute of Plant Sciences, University of Bern, Altenbergrain 21, 3013 Bern, Switzerland; Department of Biology, University of Fribourg, Chemin du Musée 10, 1700 Fribourg, Switzerland; Royal Botanic Gardens, Kew, Surrey TW9 3AB, UK; Central European Institute of Technology, Masaryk University, 625 00 Brno, Czech Republic; Department of Biology, University of Fribourg, Chemin du Musée 10, 1700 Fribourg, Switzerland; Institute of Plant Sciences, University of Bern, Altenbergrain 21, 3013 Bern, Switzerland

**Keywords:** conditionally expressed genes, dosage balance, environmental stress, subgenome dominance, transposable elements, whole-genome duplication

## Abstract

The molecular underpinnings and consequences of cycles of whole-genome duplication (WGD) and subsequent gene loss through subgenome fractionation remain largely elusive. Endogenous drivers, such as transposable elements (TEs), have been postulated to shape genome-wide dominance and biased fractionation, leading to a conserved least-fractionated (LF) subgenome and a degenerated most-fractionated (MF) subgenome. In contrast, the role of exogenous factors, such as those induced by environmental stresses, has been overlooked. In this study, a chromosome-scale assembly of the alpine buckler mustard (*Biscutella laevigata*; Brassicaceae) that underwent a WGD event about 11 million years ago is coupled with transcriptional responses to heat, cold, drought, and herbivory to assess how gene expression is associated with differential gene retention across the MF and LF subgenomes. Counteracting the impact of TEs in reducing the expression and retention of nearby genes across the MF subgenome, dosage balance is highlighted as a main endogenous promoter of the retention of duplicated gene products under purifying selection. Consistent with the “turn a hobby into a job” model, about one-third of environment-responsive duplicates exhibit novel expression patterns, with one copy typically remaining conditionally expressed, whereas the other copy has evolved constitutive expression, highlighting exogenous factors as a major driver of gene retention. Showing uneven patterns of fractionation, with regions remaining unbiased, but with others showing high bias and significant enrichment in environment-responsive genes, this mesopolyploid genome presents evolutionary signatures consistent with an interplay of endogenous and exogenous factors having driven gene content following WGD-fractionation cycles.

## Introduction

Cycles of whole-genome duplication (WGD), followed by diploidization, have been pervasive during the radiation of eukaryotes, especially in angiosperms (Jiao et al. 2011; Schranz et al. 2012; van de Peer et al. 2017; One Thousand Plant Transcriptomes Initiative 2019). Counteracting WGD events that increase the number of coexisting genomes in the nucleus and initially result in all loci being duplicated, genome fractionation (i.e. gene loss) and dysploidy (i.e. reduction of chromosome number) gradually lead to genome downsizing and a return to a diploid-like state (Lynch and Conery 2000; Tank et al. 2015; Mandáková and Lysak 2018). Despite their contribution to the architecture of genomes, neither the molecular underpinnings of such “wondrous cycles” nor the evolutionary mechanisms driving the fate of duplicated genes are fully understood (Freeling et al. 2012; Wendel 2015; Soltis et al. 2016).

Assuming an overarching connection between gene expression levels and the strength of selection acting on them, the differential expression of genes between subgenomes resulting from WGD has been postulated to drive genome fractionation by promoting the adaptive retention of specific duplicates against the accumulation of deleterious mutations and pseudogenization (Freeling 2009; Koonin and Wolf 2010). Following WGD, constraints due to the necessary dosage balance of interacting gene products are thus expected to promote the long-term retention of numerous duplicated genes with conserved functions (Birchler and Veitia 2012), whereas the partitioning of ancestral expression patterns between duplicates (i.e. subfunctionalization) supports their retention under purifying selection. In contrast, the evolution of novel functions or expression patterns is promoted by positive selection (i.e. neofunctionalization; Birchler and Yang 2022). Despite the null hypothesis that duplicated subgenomes undergo similar rates of sequence turnover, many studies have highlighted that one subgenome (coined as dominant) commonly retains more genes following WGD and is therefore “least fractionated” (LF) compared with the other subgenome(s) that appear more degenerated with fewer genes and are described as the “most fractionated” (MF) (e.g. Chalhoub et al. 2014; Garsmeur et al. 2014). Although such biased fractionation is commonly regarded as nonrandom, underlying processes remain elusive and rely on partially overlapping hypotheses of genome-wide dominance against loci presenting lower expression (Woodhouse et al. 2014; Alger and Edger 2020). In particular, interspersed copies of transposable elements (TEs) are expected to reduce the expression of nearby genes (Hollister et al. 2011) and have been predicted to influence subgenome-wide expression levels, determining dominance and patterns of biased fractionation between genomes with unbalanced TE loads (Freeling et al. 2015). Although an association between TE abundance and subgenome dominance has been documented in recently formed as well as ancient polyploid genomes (e.g. Garsmeur et al. 2014; Edger et al. 2017), several counterexamples indicate that other factors may also be at play (Douglas et al. 2015; Renny-Byfield et al. 2015; Zhao et al. 2017). Furthermore, the parental legacy of TEs associated with gene expression levels was recently shown to be insufficient to explain subgenome-wide dominance in experimental allotetraploids of *Brassica* (Zhang et al. 2023).

Beyond endogenous genomic features, exogenous factors such as different environmental conditions may also be involved in promoting the differential expression of loci between subgenomes (e.g. Shimizu-Inatsugi et al. 2017) and result in biased fractionation. The possible interactions between transcriptional plasticity in response to environmental changes and genome fractionation are indeed virtually unknown (Blischak et al. 2018).

The mustard family (Brassicaceae) that includes the model plant *Arabidopsis thaliana* contains numerous examples of taxa having undergone multiple rounds of WGD and thus offers pertinent model systems to investigate the drivers and consequences of post-polyploid genome fractionation (Kagale et al. 2014; Mandáková et al. 2017; Hendriks et al. 2023). On top of the family-specific paleotetraploidy event (α-WGD) that occurred some 32 million years ago (mya; Hohmann et al. 2015) and left several duplicated genes in all extant diploid genomes of Brassicaceae, the genus *Biscutella* comprising more than 50 species of annual herbs or perennial shrublets has rapidly radiated across the Mediterranean basin following a presumably shared mesotetraploidy event (Geiser et al. 2016). Although this WGD event was associated with hybridization between two closely related, structurally similar genomes and resulted in a functionally redundant ancestral karyotype of *Biscutella* (Guo et al. 2020), a comparative chromosome painting, coupled with transcriptomics in buckler mustard (i.e. *Biscutella laevigata*; *x* = 9), has shown that chromosomal segments conserved as duplicates are side by side with loci having undergone fractionation in this mesopolyploid (Geiser et al. 2016). Although many recently active TEs were identified in this species (Bardil et al. 2015), the lack of appropriate genomic resources to anchor genes and TEs under scrutiny to specific loci and subgenomes precluded the characterization of genome fractionation. Using an annotated chromosome-level assembly of the alpine buckler mustard genome (*B. laevigata* subsp. *austriaca*), this work thus addresses the role of endogenous TEs and environmental factors on gene expression and long-term retention. Specifically, we (i) characterized the mesopolyploid WGD event in the context of TE activity in the buckler mustard, (ii) assessed biased fractionation by comparing syntenic genes in the LF and MF subgenomes in relation to their expression, TE loads, and patterns of selection, and (iii) quantified how transcriptional changes in response to exogenous factors support the retention of duplicated genes and shape genomic regions with low versus high levels of biased fractionation.

## Results

### Assembly and Annotation of the Buckler Mustard Genome

**Fig. 1.**
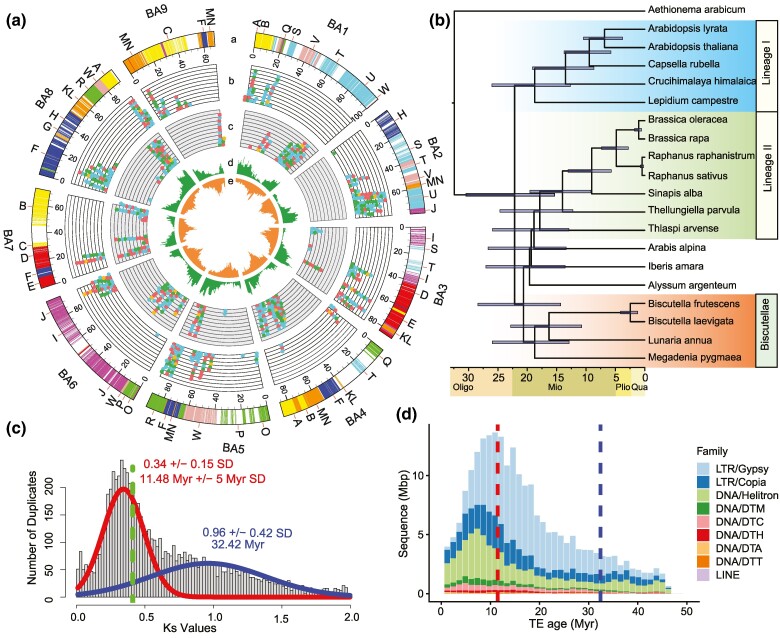
Assembly and annotation of the buckler mustard mesopolyploid genome. a) A Circos plot showing (a) the nine main scaffolds (chromosomes in Mb) of *B. laevigata* subsp. *Austriaca*, with syntenic genes shown as lines colored and labeled by capital letters according to the ancestral genomic blocks in Brassicaceae; (b) DE duplicated genes across the LF subgenome, shown as dots colored based on expression in response to environmental treatments (blue = cold, red = heat, yellow = drought and green = herbivory); (c) DEGs across the MF subgenome as in (b); (d) gene density per Mb; (e) LTR-retrotransposon density per Mb. b) Phylogenetic placement of buckler mustard among Brassicaceae and within the tribe Biscutelleae based on the analysis of whole plastid genome sequences. c) Synonymous substitutions (*K*_s_) among paralogs, with significant *K*_s_ peaks corresponding to the α-WGD event shown in blue and to the meso-WGD event in red. The green line indicates the *K*_s_-based divergence between *B. laevigata* and *A. thaliana* (see supplementary fig. S5, Supplementary Material online). d) Dynamics of the main types of TEs with dated peaks indicative of transposition bursts in relation to the mean *K*_s_ values of the α-WGD and the meso-WGD events as in (c). LTR = class I LTR retrotransposons; DNA = class II DNA transposons; DTM = mutator; DTC = CACTA; DTH = PIF-Harbinger; DTA = hAT; DTT = Tc1-Mariner; LINE = class I long interspersed nuclear element.

The allogamous sample of buckler mustard (*B. laevigata* subsp. *austriaca*) here sequenced with a combination of long and short reads (total coverage of 282×; supplementary table S1, Supplementary Material online) was estimated to have a haploid genome size of 904 Mb based on the flow cytometry analysis. *K*-mers estimated a total size of 832 Mb and a moderate heterozygosity rate of 1.88% to 1.92%, which is in between the Col-0 accession of *A. thaliana* (0.22%; Kang et al. 2023) and the T16 accession of *Brassica oleracea* (up to 5.78%; Li et al. 2024), and matches estimates for typically outcrossing diploid plants such as *Arabidopsis lyrata* (i.e. 1.4% to 2.1%; Ross-Ibarra et al. 2008) as well as the sampled population of *B. laevigata* (i.e. 3.3%; Grünig et al. unpublished). Following long-range scaffolding (supplementary fig. S1, Supplementary Material online), collapsing of similar scaffolds to reduce heterozygosity, while retaining duplicates arising from WGD events (supplementary fig. S1c, Supplementary Material online), and gap filling, a final assembly consisting of 6,350 scaffolds with a total length of 873.75 Mb (https://genomevolution.org/coge/GenomeInfo.pl?gid=67230; N50 = 71.38 Mb; supplementary fig. S2, Supplementary Material online) was produced, showing 98.2% of complete BUSCO genes (i.e. 78.1% single copy and 20.1% duplicated; supplementary table S2, Supplementary Material online). The Hi-C contact map was manually curated and scaffolded into chromosomes that showed a band of high contact density along their diagonal, reflecting the well-ordered underlying assembly submitted in supplementary fig. S2b and dataset S1, Supplementary Material online. Synteny comparisons with the ancestral genomic blocks of Brassicaceae (Lysak et al. 2016) and comparative chromosome painting confirmed the structure of the 13 largest scaffolds and supported their arrangement into the nine nearly complete chromosomes (total length: 764.46 Mb; Fig. 1a). The chromosome structures of Ba5 and Ba6, characterized by the combination of genomic blocks O + P + W + R and an inactive paleocentromere (supplementary fig. S3, Supplementary Material online), align with chromosome AK6/8 of the ancestral Proto-Calepineae Karyotype (*n* = 8), identified as the common ancestor of the tribe Biscutelleae (Guo et al. 2020). With its largely duplicated blocks, the assembly presented significant synteny with the closely related *B. laevigata* subsp. *varia* (Geiser et al. 2016), including two interstitial 5S rDNA loci identified at the pericentromeric heterochromatin of chromosomes Ba1 and Ba4, and two terminal 35S rDNA loci on chromosomes Ba2 and Ba3 (supplementary fig. S3, Supplementary Material online).

Annotated TEs comprised 539.14 Mb, encompassing 68.63% of the nine chromosome-scale scaffolds (supplementary table S3, Supplementary Material online), with a majority (ca. 40%) of long-terminal repeat (LTR) retrotransposons. Among them, a total of 2,993 full-length copies were identified (supplementary table S4, Supplementary Material online) among the main lineages of Copia (e.g. 864 Ale, 301 Ivana) and Gypsy (e.g. 293 Athila, 21 Tekay), which are predicted to have been active recently in Brassicaceae (Zhou et al. 2021). The distribution of TEs supported the structure of the assembly, with a higher abundance of LTR retrotransposons located toward the centromeric regions (Fig. 1a). Two centromeric tandem repeats were identified (213 and 468 bp; supplementary fig. S3, Supplementary Material online). Ab initio–predicted genes supported by RNA-seq data from seven tissues under mesic environments, as well as from leaf tissue of clone plants subjected to cold, drought, heat, and herbivory, resulted in the high-quality annotation of 43,632 gene models and 86.3% of the complete BUSCO genes. Despite the basal split of Biscutelleae from other Brassicaceae clades, here dated to be c. 20 mya based on the plastid phylogeny (Fig. 1b), at least 13,221 orthogroups (23,247 genes) presented a clear ortholog in all or all-but-one of the representative genomes of Brassicaceae (supplementary fig. S4 and table S5, Supplementary Material online). Together with 864 such orthogroups being absent from the *B. laevigata* assembly, these genes were classified as conserved orthologs across Brassicaceae.

### Main Molecular Drivers of Mesopolyploid Genome Evolution

The mesopolyploid nature of the buckler mustard genome seen in the structure of the assembly and the karyotype was confirmed by a peak in the distribution of synonymous substitutions (*K*_s_) among paralogs at 0.34 (±0.15 standard deviation [SD]) in addition to the peak around 0.96 (±0.42) that is indicative of the α-WGD event shared with *A. thaliana* (Fig. 1c, supplementary fig. S5, Supplementary Material online). Consistent with prior estimates, the younger meso-WGD event was dated to be younger than 11.5 mya. To assess whether it promoted the concomitant activation of TEs according to the genome-shock hypothesis or subsequently supported effective transposition due to relaxed selection (Parisod et al. 2010), we dated the amplification of TE copies by estimating their divergence from consensus sequences in the main TE lineages of the buckler mustard genome (Maumus and Quesneville 2014). Identified peaks indicative of transposition bursts were observed to range from 10% to 6% sequence divergence and indicated the ongoing transposition of several LTR retrotransposons between 10 and 5 mya (Fig. 1d). The lower divergence of TE copies than duplicated genes is consistent with a more recent onset of TE proliferation than the mesopolyploid WGD event and hence high TE dynamics during the early stages of genome fractionation in *B. laevigata*.

**Fig. 2.**
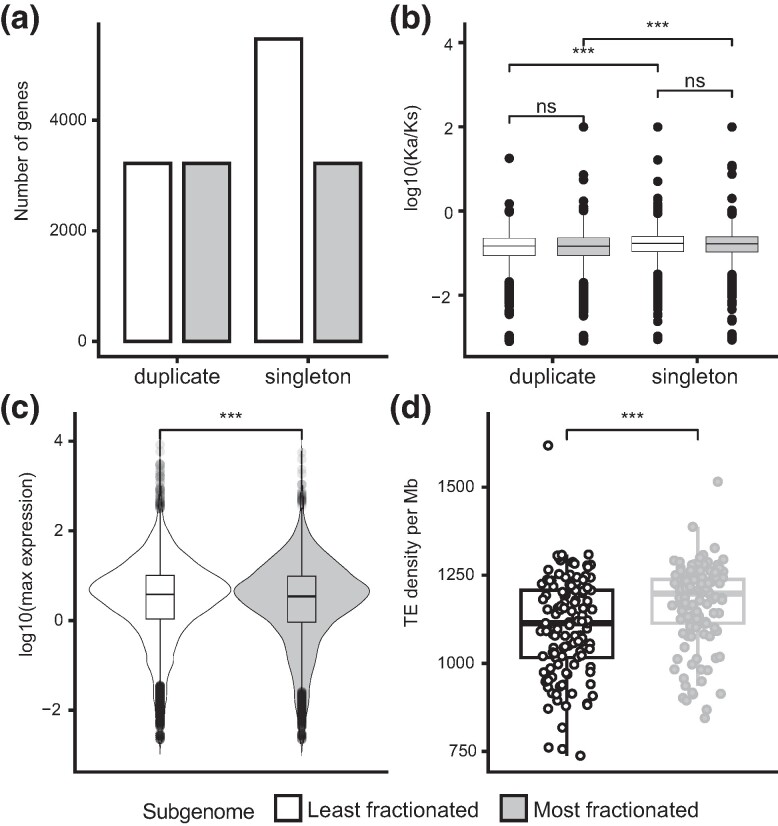
Patterns of fractionation in the LF and the MF subgenomes of the mesopolyploid buckler mustard. a) Number of retained duplicates and genes that have returned to singleton state following WGD among the 122 hi-confidence duplicated segments, showing biased fractionation with a higher number of intact genes in the LF than on the MF subgenome. b) Nonsynonymous (*K*_a_) per synonymous (*K*_s_) substitutions showing stronger signals of purifying selection among retained duplicates than singletons in each subgenome. c) Maximum expression levels of genes in leaf transcriptomes under cold, heat, drought, and herbivory treatments showing significantly higher levels of expression in the LF than in the MF subgenome. d) Density of TEs in base pairs per Mb showing a significantly lower TE load in the LF compared with the MF subgenome. Significance of Wilcoxon test represented by adjusted *P*-value <0.001 (***) and nonsignificant (ns).

Synteny between the *B. laevigata* assembly and sequences of *A. thaliana* identified 14,923 genes retained in the high-confidence chromosomal segments of syntenic duplicates derived from the meso-WGD event among a total of 122 nonoverlapping windows spanning 81.2% of the nine main scaffolds (i.e. chromosomes; supplementary fig. S6, Supplementary Material online). Downstream analyses were performed on these syntenic windows, which were shown to be mostly <2 Mb (median length 1.58 Mb; supplementary fig. S6b, Supplementary Material online), to ensure that our conclusions were largely unaffected by potential misassemblies that were shown to possibly span genome segments ≥7 Mb (supplementary fig. S2c, Supplementary Material online). These duplicated windows included 6,436 duplicates in 3,218 pairs (Fig. 2a, supplementary table S6, Supplementary Material online) that were assigned to the LF and MF subgenomes based on corresponding gene trees with high node support (supplementary fig. S7, Supplementary Material online). Beyond these retained duplicates representing 43.1% of the genes among syntenic windows, the LF and MF subgenomes presented a total of 8,542 and 6,381 intact genes, respectively, supporting differential gene retention which is consistent with biased fractionation.

To further assess the underpinnings of genome evolution, we turned to expression data and confirmed the predicted association between expression and selection by showing that expressed genes had lower ratios of nonsynonymous (*K*_a_) per synonymous substitution rates (*K*_s_) than genes considered to be unexpressed (Wilcoxon test, *P* < 0.001). The maximum level of gene expression was shown to be linearly associated with *K*_a_/*K*_s_ values (slope = −0.10, *P* < 0.001, supplementary fig. S8, Supplementary Material online), matching the expectation that highly expressed genes are conserved under stronger purifying selection (i.e. lower values of *K*_a_/*K*_s_). The majority of genes under scrutiny in the buckler mustard indeed showed conserved coding sequences under pervasive purifying selection (i.e. 14,745 genes with *K*_a_/*K*_s_ < 0.79 when compared with *A. thaliana*). Despite similar signals of purifying selection across both subgenomes, pairs of duplicated genes presented significantly lower *K*_a_/*K*_s_ ratios than genes that had returned to singleton state and this difference was particularly pronounced in the LF subgenome (Fig. 2b; *P* < 0.001).

Addressing how expression and selection shaped gene retention between subgenomes, we characterized the expressed genes in the LF (6,771) compared with the MF subgenome (4,920; *z*-test, *P* < 0.01) and showed significantly higher gene expression in the LF than in the MF subgenome (Wilcoxon test, *P* < 0.001; Fig. 2c and supplementary fig. S9, Supplementary Material online). As expected, expressed genes in the MF subgenome accordingly showed stronger signals of purifying selection than those in the LF subgenome (average *K*_a_/*K*_s_ of 0.170 and 0.175, respectively; Wilcoxon test, *P* < 0.01), suggesting that high gene expression associated with strong purifying selection has been necessary to support gene retention in the MF subgenome.

In contrast to genes, the density of TEs was significantly lower in the LF subgenome than in the MF subgenome (Wilcoxon test, *P* < 0.001; Fig. 2d). Given that the presence of TE copies up to 2,000 bp upstream or downstream of genes was significantly associated with their lower levels of expression (supplementary fig. S10 and table S7, Supplementary Material online), such differential TE load likely played a role in shaping gene expression and hence selection across the LF and MF subgenomes. The presence of a TE copy within 201 to 2,000 bp was seen to reduce the median expression of nearby genes by 28.14% (1.83 log_2_-fold change), as expected by their epigenetic silencing locally affecting flanking loci. Although such indirect effects of TEs may have contributed to the long-term-biased fractionation under pervasive purifying selection, the observed association between TE density and gene retention does not exclude other drivers of fractionation or other triggers of gene expression across subgenomes as drivers of fractionation.

### Environmental Triggers of Duplicate Retention Following WGD

**Fig. 3.**
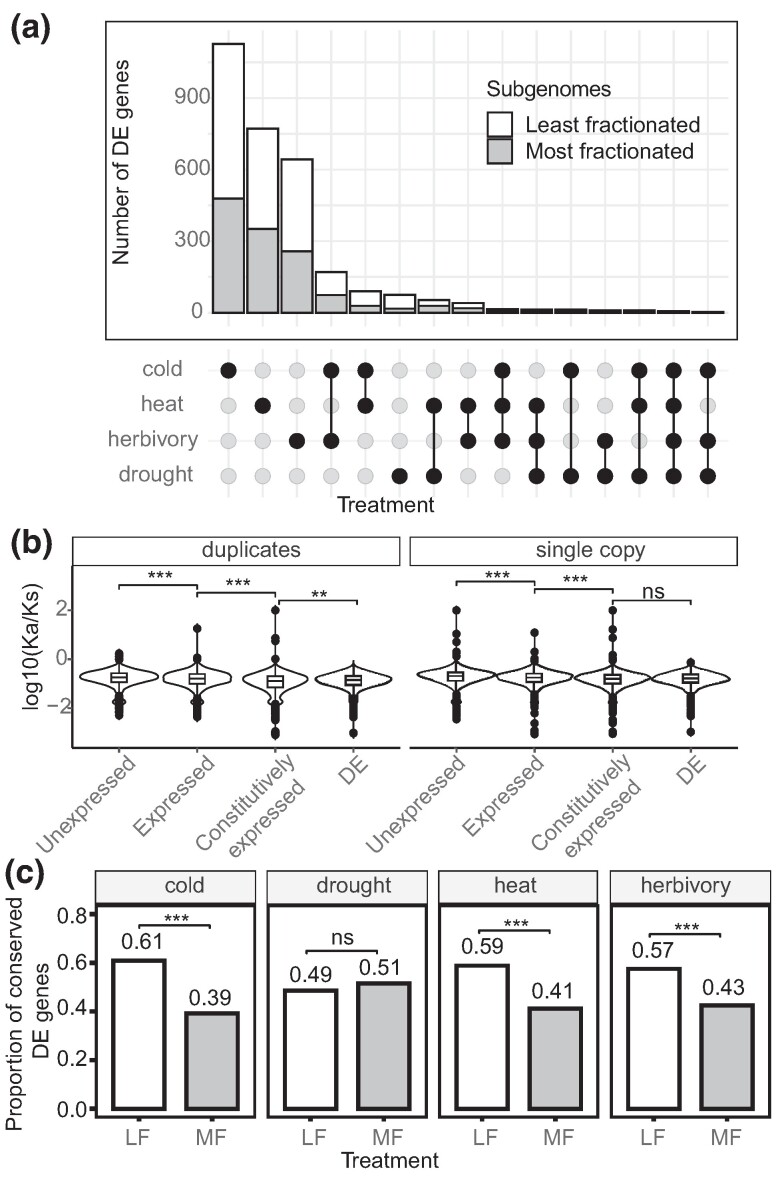
Expression and selection of environmentally responsive genes in the mesopolyploid buckler mustard. a) Distribution of DEGs in response to cold, heat, drought, and herbivory treatments. b) Patterns of selection based on *K*_a_/*K*_s_ values between duplicated and single-copy genes according to their type of expression, showing stronger conservation of coding sequences among environmentally responsive (DE) genes and constitutively expressed genes compared with unresponsive genes (i.e. genes that were either unexpressed or were expressed but were not DE). Significance of Wilcoxon tests represented as adjusted *P*-value <0.001 (***), < 0.01 (**), and nonsignificant (ns). c) Proportions of DE genes conserved across Brassicaceae showing biased distribution across the LF when compared with the MF subgenome. Significance of Fisher's exact tests represented as adjusted *P*-value <0.001 (***) and <0.01 (**).

To what extent conditional gene expression in response to environmental cues has been shaping genome fractionation was first assessed by inspecting the 977 single-copy and 760 duplicated genes from 966 pairs that were differentially expressed (DE) in response to experimental cold, heat, drought, and herbivory treatments out of the 14,923 syntenic genes under scrutiny in the LF and MF subgenomes (Fig. 3a; supplementary fig. S10, Supplementary Material online). Noticeably, the *K*_a_/*K*_s_ ratios of DE genes were significantly lower than for genes with other expression patterns (Wilcoxon tests, *P* < 0.001; see Fig. 3b), indicating that coding sequences conditionally expressed in response to environmental cues were more likely to be retained under stronger purifying selection than constitutively expressed ones. It is notable that the patterns of selection in DE-duplicated genes were consistent with retention under even stronger purifying selection than DE single-copy genes (i.e. *K*_a_/*K*_s_ of 0.155 and 0.176, respectively; Wilcoxon test, *P* < 0.001), indicating that abiotic and biotic cues promoted the long-term adaptive retention of duplicates in this mesopolyploid genome. Matching the genome-wide pattern of fractionation, retained DE genes were significantly more numerous in the LF than the MF subgenome, as shown for conserved orthologs across Brassicaceae in Fig. 3c.

By cross-matching patterns of expression and signals of selection in the buckler mustard, we assessed evolutionary underpinnings of 2,196 expressed pairs of retained duplicates across syntenic windows, considering their orthologs in *A. thaliana* as “progenitor singletons” (supplementary table S6, Supplementary Material online). Consistent with the advanced fractionation of the mesopolyploid genome, only eight pairs of duplicated genes (0.36%) presented signals of neutral divergence (0.79 < *K*_a_/*K*_s_ ≤ 1.21) for either both or one member of the pair and were hence possibly retained without selection. Only four pairs of duplicates (0.18%) presented one member with a clear signal of positive selection (*K*_a_/*K*_s_ > 1.21) pointing to possible neofunctionalization, while the other member was retained under purifying selection.

In contrast, the vast majority of expressed duplicates retained in the buckler mustard presented both copies under purifying selection (99.4%). Considering their expression in response to cold, heat, drought, and/or herbivory treatments to highlight possible changes in the environmental trigger(s) compared with their progenitor singletons, almost two-thirds of the retained duplicates in the buckler mustard showed either no environmental trigger (1,367 pairs) or were both DE under similar conditions compared with their progenitor singletons (33 pairs) and were thus considered consistent with retention of conserved function under dosage balance constraints (63.8% of expressed duplicates). The remaining third of duplicates retained under purifying selection (796 pairs; 36.2%) showed a change in response to an environmental trigger compared with the progenitor singletons. A total of 296 duplicate pairs showed both members having lost their ability to respond to the environmental trigger(s), whereas 27 and 31 pairs presented one member with a conserved environmental trigger on the LF and MF subgenomes, respectively, with the other member being constitutively expressed. Nevertheless, the majority of the expressed duplicates (i.e. 391 pairs) presented gain(s) in environmental trigger(s) in the buckler mustard, affecting either one or both members and indicating considerable expression repatterning during fractionation.

Pointing to dosage balance as the chief constraint driving the retention of duplicated genes, our results also highlight the importance of expression changes among environment-responding duplicates following fractionation. Expression changes chiefly resulted in the constitutive expression of one or both member(s) and hence increased dosage of conserved coding sequences that were ancestrally stress responding among retained duplicates in the buckler mustard. A similar evolutionary response of constitutive expression was apparent beyond the duplicated segments under scrutiny here, with most of the 2,098 progenitor singletons previously shown to be environmentally responsive in *A. thaliana* being identified as singleton (1,032, 49.2%) or retained as duplicate (353) presenting constitutive expression in the buckler mustard. Among the 222 progenitor singletons retained as environment-responsive duplicates in *B. laevigata*, only 57 had both members responding to at least 1 environmental treatment, while 70 presented constitutive expression of 1 member. Such canalization of ancestrally environment-responsive genes toward constitutive expression is consistent with the “turn a hobby into a job” model (Conant and Wolfe 2008) and likely promoted the increased tolerance of the buckler mustard to the stressful conditions that are typical of alpine environments where it currently thrives.

### Biased Retention of Environment-Responsive Genes During Genome Fractionation

**Fig. 4.**
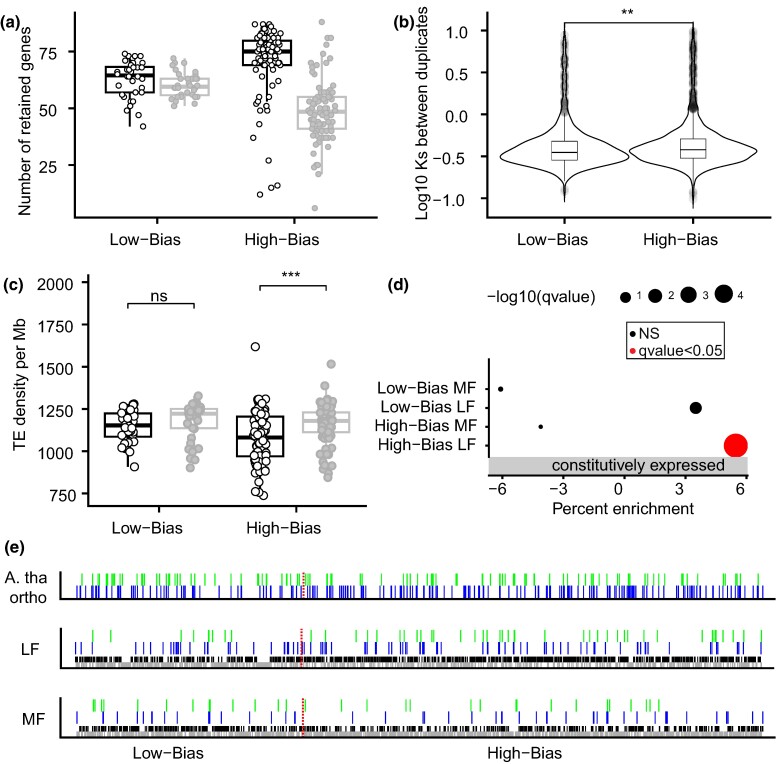
Biased retention of environment-responsive genes across regions of the LF versus the MF subgenomes. a) Analysis of the 122 windows partitioned into 36 low-bias and 86 high-bias regions showing similar versus significantly different numbers of retained duplicate genes between the LF (open circles) and MF (filled gray circles) subgenomes, respectively. b) Divergence based on synonymous substitutions (*K*_s_) between duplicated genes in the low-bias versus high-bias fractionation regions. c) Density of TEs in base pairs per Mb in the LF and MF subgenomes is not significantly different in low-bias regions, whereas the high-bias regions of the MF subgenome have significantly higher TE density than the LF subgenome. d) Enrichment of gene set when compared with the whole genome showing constitutively expressed genes are significantly over-represented in the high-bias LF regions. e) Genomic segment (genomic block U) showing a pattern of biased retention of genes responding to cold (depicted in blue) and herbivory (depicted in green) within the high-bias LF subgenome. Constitutively expressed genes are represented in black, while lost or unexpressed genes are depicted in gray. The first panel illustrates differentially expressed genes related to cold and herbivory in *A. thaliana* (labeled A. tha ortho), whereas the second and third panels show the LF and MF subgenomes in *B. laevigata*, respectively. The dashed red line delineates the segment into its low-bias and its high-bias region. Significance of Wilcoxon tests represented as adjusted *P*-value <0.001 (***), <0.01 (**), and nonsignificant (ns).

To further assess how the environment-responding genes evolved across subgenomes, we partitioned the 122 windows of syntenic duplicates into (i) 36 regions comprising those with “low-bias” (i.e. regions with nonsignificant differences in the proportion of retained genes between the LF and the MF subgenomes), and (ii) 86 “high-bias” regions (i.e. regions characterized by a significantly reduced proportion of retained genes in the MF subgenome compared with the LF subgenome; *χ*^2^ test, *P* < 0.05; Fig. 4a). Low-bias regions were shown to contain duplicated genes characterized by significantly lower divergence than in high-bias regions (Fig. 4b) and they did not differ in TE density, unlike high-bias regions (Fig. 4c). These low-bias regions are therefore considered to have undergone limited TE-driven-biased fractionation and possibly may have had prolonged exchanges between subgenomes that supported unbiased fractionation in the absence of differential TE load. Overall, high-bias regions of the LF subgenome were significantly enriched in constitutively expressed duplicates (Fig. 4d).

The retention of genes responding to environmental triggers was otherwise consistent across high-bias regions, showing pervasive conservation of DE and constitutively expressed genes on the LF subgenome. Biased fractionation hence supported the retention of specific environment-responding genes, as seen across the genomic block U (Fig. 4e) that is duplicated on chromosomes Ba1 and Ba2 and whose high-bias region on the LF subgenome appeared significantly enriched in DE genes responding to cold (Gene ratio: 58/724, *q* < 0.01; supplementary table S8, Supplementary Material online) and herbivory (Gene ratio: 36/724, *q* < 0.01). Genes related to the isopentenyl diphosphate biosynthetic process (GO:0019288, comprising 15 genes; supplementary table S9, Supplementary Material online) were notably abundant across that segment of the LF subgenome showing an enrichment of KEGG terms related to plant–pathogen interactions (comprising 8 genes; supplementary table S10, Supplementary Material online) and suggesting specialization of the locus in terpenoid biosynthesis such as reported in a previous study of the buckler mustard (Knauer et al. 2018). Genome fractionation in such regions is hence biased toward the retention of genes essential for survival under harsh conditions that only polyploids can harness through an abundance of gene copies being adaptively sorted (van de Peer et al. 2021).

Among the 14,085 orthogroups conserved in all but one of the considered Brassicaceae species, 93.8% were present in *B. laevigata*, supporting the necessary presence of most genes in each progenitor genome. Among the syntenic windows, the LF subgenome indeed presented significantly more of these conserved genes (i.e. 58.0%) than the MF subgenome (i.e. 42.0%; *P* < 0.001, Fisher's exact test) and a similar enrichment was also reflected among singletons (i.e. 64.1% in the LF subgenome compared with 51.7% in the MF subgenome; *P* < 0.001, Fisher's exact test). This pattern held true for the specific genomic block U, in which the LF subgenome harbored a significantly higher proportion of conserved genes (59.1%, *P* < 0.001, Fisher's exact test), among which were a higher percentage of singletons (60.3%) compared with the MF subgenome (45.9%, *P* < 0.001, Fisher's exact test). Such overall and locus-specific enrichments of conserved duplicates and singletons in the LF subgenome strongly support that progenitor genomes have contributed similar sets of genes and, despite possible subtle differences in their regulatory circuits before hybridization, had undergone post-WGD sorting that chiefly shaped the two subgenomes of *B. laevigata*.

## Discussion

The mesotetraploid genome of the buckler mustard originated by WGD coupled with hybridization between two closely related progenitors which contributed similar gene sets before the polyploid genome started to undergo diploidization over about 11.5 million years in association with descending dysploidy toward nine pairs of chromosomes (Geiser et al. 2016; Guo et al. 2020). Despite bioinformatic challenges arising from aiming to reduce heterozygosity while maintaining WGD-derived duplicates in a haploid assembly and those inherent to reconstructing the history of polyploids and their long-extinct progenitors (Kellogg 2016), the outcomes of long-term-biased fractionation are still visible across the majority of 122 duplicated segments in the mesotetraploid genome today. Although our results appear consistent with predictions of TE-driven subgenome dominance (Alger and Edger 2020), 36 of these duplicated segments (27.0%, spanning 241 Mb) actually show unbiased fractionation suggestive of locus-specific rather than (sub)genome-wide drivers. Further, contrasting with the legacy of progenitor TEs determining subgenome dominance, our data show that the proliferation of several types of TEs took place during the early stages of genome fractionation in the buckler mustard and instead support the prediction that relaxed selection on the initially redundant loci cumulatively fostered the biased genomic divergence toward an LF versus MF subgenomes (Woodhouse et al. 2014; Bird et al. 2018). Although the exact TE composition of the long-extinct progenitors is unknown and their role in driving subgenome dominance immediately after WGD remains elusive, the partially biased fractionation of the mesopolyploid genome of *B. laevigata* appears consistent with runaway pseudogenization coupled with the loss of lowly expressed genes that could only have been antagonized by strong selection resulting in the retention of highly expressed genes, including duplicates mostly constrained by dosage balance (Blanc and Wolfe 2004).

Here, we show that, in addition to the role of endogenous factors such as TEs and genes involved in dosage balance, exogenous factors, i.e. different environmental conditions driving the conditional expression of genes (as shown in some previous studies; e.g. Shimizu-Inatsugi et al. 2017; Lee and Adams 2020), have also substantially contributed to genome fractionation. Despite the many challenges inherent to distinguishing the partitioning of ancestral functions from gain(s) of novel environmental triggers (Innan and Kondrashov 2010; Birchler and Yang 2022), numerous ancestral environment-responsive genes with conserved coding sequences were identified as having promoted increased dosage through the evolution of constitutive expression and/or the retention of duplicates as a pervasive outcome of long-term fractionation. Although expression changes can be expected to evolve neutrally through time (Khaitovitch et al. 2004), transcriptional plasticity in response to environmental conditions was generally retained by only one member of the duplicate pair, with the other showing constitutive expression that likely supported general survival under stressful environmental conditions (Conant and Wolfe 2008; van de Peer et al. 2021). While such co-option of transcriptionally plastic genes that promoted constitutive adaptation to exogenous factors may have been instrumental in shaping the current mesopolyploid genome, it likely imposed costs and hence may contribute to explaining the slow growth of the perennial *B. laevigata* under alpine conditions. Connections between WGD per se and stressful conditions in the short-term remain elusive, although insights from our analysis of buckler mustard's mesopolyploid genome point to post-WGD fractionation and particularly the retention of environment-responsive duplicates coupled with expression changes as key to their possible radiation across harsh environments (Dodsworth et al. 2016). Although here we have unraveled plausible mechanisms linking WGD and increased stress tolerance that have operated over millions of years of evolution, future work using experimentally resynthesized and recently established polyploids will be needed to address how genome fractionation unfolds through time and affects the fate of duplicated genes from the initial WGD event to the highly fractionated mesopolyploid genomes entering new rounds of WGD (Soltis et al. 2016; Bird et al. 2020; Parisod 2024).

## Materials and Methods

This section gives a summary of the methodology, which is detailed in the supplementary methods, Supplementary Material online.

### Plant Material, Sequencing, Assembly, and Annotation

The same individual sample of *B. laevigata* subsp. *austriaca* grown from a seed collected near Schneealpe (Steiermark, Austria: 47.6968°N, 15.6100°E; 1,740 m asl) was used throughout, from de novo genome assembly to RNA-seq data, using regenerated cuttings (i.e. clonal ramets).

The genome size was estimated by flow cytometry, and high-molecular-weight DNA was sequenced with short Illumina 10× genomics linked reads (75×) which has been shown to produce reliable assembly in maize, a species that also went through WGD some 5 to 12 mya (Visendi 2022). Linked reads dataset was complemented with a combination of long Pacbio reads (12×) of an average length of 5.3 kb, and paired-end reads (75×; supplementary table S1, Supplementary Material online). The hybrid assembler Platanus-allee, which marks better performance in highly heterozygous genomes (Kajitani et al. 2019), produced a draft genome that was scaffolded by using Chicago (52× coverage) and Hi-C (68× coverage) methods (Dovetail Genomics, Santa Cruz, CA, USA). Hi-C maps may contain errors or inaccuracies that were carefully evaluated and, combined with evidence from cytogenetic maps, refined to ensure a more accurate genomic assembly. *K*-mers (*k* = 21) were counted using Jellyfish, and the resulting histogram was analyzed with GenomeScope2 (Ranallo-Benavidez et al. 2020) to estimate genome size, heterozygosity, and repeat content. After removal of uncollapsed haplotigs and gap filling, the completeness of the final assembly was assessed with the BUSCO from embryophyte odb10. Merqury (Rhie et al. 2020) was then employed to compare the heterozygous *k*-mer content before and after removal of uncollapsed haplotigs. Curation of the 13 largest scaffolds into the 9 main chromosome-level scaffolds was further validated through comparative chromosome painting, as described in Geiser et al. (2016).

Repetitive elements across the assembly were first identified based on TE structural features using EDTA (Ou et al. 2019). The dynamics of TEs were estimated based on the percentage of divergence of each copy to the consensus according to Maumus and Quesneville (2014) and dated using 8.22 × 10^−9^ substitutions/synonymous site/year for Brassicaceae species (Kagale et al. 2014).

Genes were annotated using ab initio and mapped RNA-seq reads from seven tissues (i.e. roots, young leaves, senescent leaves, stems, apical meristem, floral buds, and open flowers; European Nucleotide Archive accession: PRJEB48599) and leaf tissues under different environmental conditions (see *Gene expression in response to environmental changes* section) as well as Swissprot protein sequences from Viridiplantae used as homology-based support. Only annotations with an edit distance <0.5 and coding for proteins >20 amino acids were considered.

### Gene Expression in Response to Environmental Changes

Replicated leaf transcriptomes in response to environmental treatments (European Nucleotide Archive accession: PRJEB48469) were generated from clones of the sequenced individual subjected to control (22 °C, 16/8 h light/dark cycle), cold (24 h at 4 °C, 16/8 h light/dark), heat (3 h gradual increase from 22 to 42 °C and 6 h at 42 °C), drought (11.5 d without watering) and herbivory condition (30 h of feeding by the moth *Plutella xylostella*). Those treatments were designed to mimic data available for *A. thaliana* (Klepikova et al. 2016; Dubois et al. 2017; Nallu et al. 2018) as the only other plant species whose transcriptional responses to several environments has been investigated.

Gene expression was quantified using RSEM (Li and Dewey 2011), with only genes expressed at >1 transcript per million considered as “expressed.” DE genes (DEGs) presenting a log_2_-fold change >1 were identified using edgeR (Robinson et al. 2010).

### Analysis of Duplicated Chromosome Segments

The “SynMap” algorithm within CoGe (https://genomevolution.org/CoGe/GEvo.pl) identified duplicated genes from the mesopolyploid WGD event through collinearity within the buckler mustard genome and with ancestral genomic blocks of Brassicaceae extracted from *A. thaliana*. Following Woodhouse et al. (2011), windows of syntenic duplicates were seeded with ten collinear genes, comprising 100 *A. thaliana* genes and corresponding duplicates of the buckler mustard genome, with a maximum of 20 nonsyntenic genes to be considered.

Windows of syntenic duplicates were assigned to the two subgenomes according to RAxML phylogenetic trees of orthologous coding sequences from *A. thaliana*, *Megadenia pygmaea*, and the sister genus *Heldreichia bupleurifolia* and accordingly classified as LF and MF following Guo et al. (2020).

SynMap further determined synonymous substitution rates (*K*_s_) and nonsynonymous substitution rates (*K*_a_) when compared with *A. thaliana* orthologs. Approximate Gaussian distributions of *K*_s_ between duplicates marking WGD events were detected by mixture models using mixtools (https://github.com/dsy109/mixtools). The α-WGD event (mean *K*_s_ = 0.96) dated at 32.42 mya (Hohmann et al. 2015) was used as a calibration point to estimate the minimum age of the mesopolyploid WGD event.

A signal of selection was assessed using *K*_a_/*K*_s_ values when compared with orthologous loci in *A. thaliana*, considering genes to be under purifying selection when *K*_a_/*K*_s_ ≤ 1 − SD (i.e. ≤ 0.787), neutral when 1 − SD < *K*_a_/*K*_s_ ≤ 1 + SD, and under positive selection when *K*_a_/*K*_s_ > 1 + SD (i.e. >1.213).

Duplicated windows from each subgenome were further partitioned into low-bias regions presenting quasi-unbiased fractionation (i.e. with a similar number of retained syntenic genes in the MF and LF subgenomes) and high-bias regions undergoing heavily biased fractionation (i.e. a significantly different number of retained syntenic genes between the MF and the LF subgenomes) based on *χ*^2^ tests (nonsignificant difference in the proportion of retained genes in MF and LF (*P* > 0.05) classified as low bias and significant difference classified as high bias).

## Supplementary Material

msae155_Supplementary_Data

## Data Availability

Raw sequence data are available on the European Nucleotide Archive repository (https://www.ebi.ac.uk/ena/browser/home), as follows: Genomic DNA sequence data: PacBio long reads (SRR26423064), Paired-end Illumina short reads (SRX8787129), 10× genomics-linked reads (SRX8815186), Chicago Illumina short reads (SRR26396391), and Hi-C Illumina short reads (SRR26404274). Transcriptomic data: RNA seq among tissues (ERP132985) and RNA seq among environmental treatments (ERP132838). The genome assembly is available at: https://genomevolution.org/coge/GenomeInfo.pl?gid=67230.
