# Supplementary material for: Biased Retention of Environment-Responsive Genes Following Genome Fractionation
Source: Mol Biol Evol. 2024 Jul 29;41(8):msae155. doi: 10.1093/molbev/msae155 (PMC11306978; doi:10.1093/molbev/msae155)
Supplement: msae155_Supplementary_Data [file msae155_supplementary_data.zip › Beringer_Choudhury_etal_SI.pdf]

Supporting Information for

Biased retention of environment-responsive genes following genome fractionation

Marc Beringer, Rimjhim Roy Choudhury, Terezie Mandáková, Sandra Grünig, Ilia J. Leitch, Martin A. Lysak, Christian Parisod

Corresponding author: Christian Parisod

Email: christian.parisod@unifr.ch.

This PDF file includes:

Supporting text, extended Material and Methods

SI References

Figures S1 to S10

Other supporting materials for this manuscript include the following:

Tables S1 to S10, in a separate .xl file

Supplementary Methods

Plant material, DNA content and sequencing

The same individual sample of *Biscutella laevigata* subsp. *austriaca* grown from a seed collected near Schneealpe (Steiermark, Austria: 47.6968°N, 15.6100°E; 1740 m asl) was used throughout, from *de novo* genome assembly to RNAseq data, using regenerated cuttings (i.e. clonal ramets).

The size of the holoploid genome was estimated by flow cytometry using cell nuclei isolated from fresh leaves. One sepal and a fully developed intact leaf was prepared as described by Doležel et al. (2007). Isolated nuclei were stained using propidium iodide and RNAase IIA (both 50 µg/ml) at room temperature for 5 min and analyzed using a Partec (Franklin Park, IL, USA) CyFlow flow cytometer. The fluorescence intensity of at least 5000 particles was recorded. *Solanum pseudocapsicum* (1C = 1.30 pg; Temsch et al., 2022) served as the primary reference standard. We measured and analyzed one individual over three consecutive days.

High molecular weight DNA was isolated after a 72-h dark treatment from 1 g of frozen leaf tissue using Qiagen Genomic Tips 100/G following the manufacturer's protocol. Following further purification with 1X AMPure beads and quality control using Femtopulse (Advanced Analytical), one ng of DNA with fragments >100 kb was prepared for GEM library creation following the standard protocol outlined in the Chromium Genome Reagent Kits v2 User Guide (CG00043 Rev B; with adherence to the Chromium Genome Reagent Kits v2 User Guide Rev A to Rev B Revision Summary) along with the Chromium Genome Chip Kit v2 and the Chromium Genome Library Kit & Gel Bead Kit v2 (10× Genomics, PN-120257 and PN-120258, respectively). The post GEM and the post library construction quality control assessments were carried out using an Advanced Analytical Fragment Analyzer System with a Fragment Analyzer HS NGS Fragment Kit (1-6000bp) (Agilent, DNF-474). The library was sequenced paired-end (2 x 250 bp) using a shared Illumina NovaSeq 6000 S Prime (SP) Reagent Kit (500 cycles; Illumina, 20012865) on an Illumina NovaSeq 6000 sequencer. This produced 164,229,013 paired-end reads for this library, corresponding to more than 75X coverage (Table S1). Linked-reads were formatted using the "longranger basic" command of Long Ranger which formats barcode sequences as "BX: Z:" tag information.

PacBio library construction and sequencing in 5 SMRT cells produced over two million reads with a mean length of only 5,348 bp (Table S1). The PacBio subreads were error corrected with Illumina paired-end reads using Fmlrc v.1 and, after error correction, resulted in a coverage of PacBio data of 12× (number of reads = 2,321,789; mean length = 5,230 bp).

Illumina paired-end (insert sizes: 550 bp) library was generated using the Illumina TruSeq PCR-free kit and were sequenced on an Illumina NovaSeq 6000 sequencer, with 250 bp read lengths (Table S1). The

resulting Illumina reads were quality-trimmed and adapters were removed with Trimmomatic v0.36 with a 2-bp mismatch, a palindrome clip threshold of 30, and a simple clip threshold of 10. Reads were then filtered based on an average phred score calculated from a sliding window of 5 bp with a minimum threshold of 15. A total of 328,458,026 reads longer than 100 bp were retained, corresponding to a coverage of 75X.

Draft and chromosome-level genome assembly

Trimmed PacBio and Illumina 10x genomics linked and paired-end reads were processed through a hybrid genome assembly approach using Platanus-allee (Kajitani *et al.*, 2019) as it promotes the arrangement of two independently assembled haplotypes corresponding to the homologous chromosomes. We first calculated the best k-mer length (k-mer = 21-131 in 10 bp increments) for assembly and predicted the genome assembly size with Kmergenie v.1.7016 using the paired-end Illumina data. Quality-controlled and trimmed Illumina paired-end reads were then assembled into contigs using `platanus_allee assemble` with an initial k-mer size of 32 and k-mer extension step size of 20 to reconstruct the Bruijn graph for each incremental k values. The resulting 3,363,789 contigs (N50 = 655 bp; assembly size = 3.36 Gb) were then phased with the formatted linked-reads and the error-corrected PacBio reads using ` platanus_allee phase` with three rounds of the haplotype synteny-based assembly algorithm. Finally, the primary-bubbles and non-bubble sequences were connected, to obtain a conventional haploid consensus format of scaffolds with a total of 225,180 contigs (N50 = 24,538 Kb; total size = 1,240 Mb; 94.5% complete BUSCO genes (see below).

Dovetail Genomics (Santa Cruz, CA) scaffolded the initial hybrid genome assembly using their propriety ChicagoTM and Hi-C methods. Chicago libraries were generated following (Putnam *et al.*, 2016), from ~500 ng of high molecular weight DNA fixed with formaldehyde and digested with DpnII. The 5' overhangs were filled in with biotinylated nucleotides, whereas free blunt ends were ligated, and crosslinks were then reversed before the DNA was purified and treated to remove biotin that was not internal to ligated fragments. Sequencing libraries were then generated from sheared DNA at ~350 bp mean fragment size using NEBNext Ultra enzymes and Illumina-compatible adapters. Biotin-containing fragments were isolated using streptavidin beads before PCR enrichment of each library. The Hi-C library was prepared from chromatin fixed in the nucleus with formaldehyde and then following a similar procedure as just described (Lieberman-Aiden *et al.*, 2009). Chicago and Hi-C libraries were sequenced on an Illumina HiSeq X to produce 245 million 2x150 (i.e. 52X coverage) and 188 million 2x150 bp paired end reads (i.e. 68X coverage), respectively.

The input *de novo* assembly, shotgun reads, Chicago library reads, and Dovetail Hi-C library reads were used as input data for HiRise (Putnam *et al.*, 2016) and an iterative analysis was conducted. First, Shotgun and Chicago library sequences were aligned to the draft input assembly using a modified SNAP read mapper (http://snap.cs.berkeley.edu). HiRise then produced a likelihood model based on genomic distances between Chicago read pairs mapped within draft scaffolds that was used to identify and break putative misjoins, to score prospective joins, and make joins above a threshold. Dovetail Hi-C library sequences were then aligned and scaffolded following the same method. Finally, after scaffolding, shotgun sequences were used to close gaps between contigs, yielding an assembly with dramatically increased contiguity (N50 = 62.83 Mb; Fig. S1).

The assembly was filtered with Redundans v0.13c (Pryszcz & Gabaldón, 2016) to collapse contigs with ≥90% similarity and with >90% overlap to remove duplicated haplotypes originating from the expected high heterozygosity of wild individuals and gaps were then filled using the error corrected PacBio long reads data using TGS-GapCloser (Xu *et al.*, 2020). The completeness of the genome assembly was assessed with BUSCO v4.0.6 (Manni *et al.*, 2021) and embryophyta odb10. Following the stringent removal of redundancy, remaining duplicated BUSCO genes in final assembly of the *B. laevigata* are likely the result of ancient WGD events in the genus.

Cytogenetic analyses

Mitotic and meiotic (pachytene) chromosome spreads from fixed young flower buds containing immature anthers were prepared as described previously (Geiser *et al.,* 2016). A total of 674 chromosome-specific BAC clones of *A. thaliana* grouped into contigs corresponding to eight chromosomes and 22 genomic blocks (GB) of the Ancestral Crucifer Karyotype (Lysak *et al*., 2016) were used. See Mandáková *et al.* (2019) for delineation of the GB boundaries. The *A. thaliana* BAC clone T15P10 (AF167571) was used for *in situ* localization of 35S rDNA, and the *A. thaliana* clone pCT4.2 (M65137) was used for localization of 5S rDNA loci. The 213-bp (CL9; CTCGAGTCCACTTCCAAACACACAAACCTAGAACTTAGTCTAGCAAAGCGCAATCGAAGA) and 468-bp (CL98; TAATACTCATTGAGCTTGAGTAGATAAAACTCCTAATTGTACACTAGATTCTATAGATTA) tandem repeats were synthesized according to Dogan et al. 2021. All DNA probes were labeled by nick translation as described by Geiser *et al*. (2016), pooled according to the design of a particular experiment and precipitated by adding 1/10 volume of 3 M sodium acetate, pH 5.2, and 2.5 volumes of ice-cold 96% ethanol, kept at -20°C for 30 min, and centrifuged at 13,000 g at 4°C for 30 min. The pellet was resuspended in 20 µl of the hybridization mix (50% formamide and 10% dextran sulfate in 2×SSC) per slide. 20 µl of the probe was pipetted onto a chromosome-containing slide. The probe and

chromosomes were denatured together on a hot plate at 80°C for 2 min and incubated in a moist chamber at 37°C overnight. Post-hybridization washing and immunodetection of hapten-labeled probes was performed according to Geiser *et al*. (2016). Chromosomes were counterstained with 4', 6-diamidino-2-phenylindole (DAPI, 2 µg/ml) in Vectashield (Vector Laboratories). Fluorescent signals were analyzed and photographed using a Zeiss Axioimager epifluorescence microscope and a CoolCube camera (MetaSystems). Images were acquired individually for the four fluorochromes using corresponding excitation and emission filtres (AHF Analysentechnik). The captured images were pseudocolored and merged using Adobe Photoshop CS6 software (Adobe Systems).

Plastid phylogeny and placement across Brassicaceae

For a selection of 20 species representative of the main lineages within the family Brassicaceae (Hohmann *et al.*, 2015; Huang *et al.*, 2016), sequences of whole plastids were either retrieved from NCBI (i.e. *Arabidopsis lyrata* NC 034365.1; *Arabidopsis thaliana* NC 000932.1; *Arabis alpina* NC 023367.1; *Brassica oleracea* NC 041167.1; *Brassica rapa* NC 040849.1; *Capsella rubella* NC 027693.1; *Crucihimalaya himalaica* NC 061290.1; *Iberis amara* NC 049655.1; *Raphanus sativus* NC 024469.1; *Sinapis alba* NC 045948.1; *Thlaspi arvense* NC 034362.1) or assembled from raw sequence data (i.e. *Alyssum argenteum* ERR2560001; *Lepidium campestre* ERR2990267; *Raphanus raphanistrum* SRR954568) with GetOrganelle (Jin *et al.*, 2020). This selection further included the demonstrated outgroup of core Brassicaceae (i.e. *Aethionema arabicum* NC 034367.1) and was enriched in species from the Biscutellae tribe (i.e. *Lunaria annua* NC 049659.1; *Megadenia pygmaea* NC 034357.1; *Biscutella frutescens* ERR3507527; *Biscutella laevigata* ERR3507536).

Plastid sequences were aligned with MAFFT 7.475 (Katoh & Standley, 2013) using the default FFT-NS-2 alignment method and manually refined before molecular dating using the Bayesian approach implemented in BEAST 2.4.6 (Bouckaert *et al.*, 2019). The best-fit substitution model, determined using the online Smart Model Selection in PhyML (www.atgc-montpellier.fr/sms/) (Lefort *et al.*, 2017), was the GTR + G + I, whereas the speciation prior was modelled by a Yule process and a log-normal relaxed molecular clock was used. Enforcing monophyly of the ingroup, the age of the common ancestor rooting the tree was set to a normal distribution around the mean of 32.42, with a standard deviation of 0.0001, following (Hohmann *et al.*, 2015). Five analyses were run for 1,000,000,000 generations, sampling a tree every 50,000 generations. The convergence of the runs and the appropriate burn-in period (i.e. from 100,000,000 to 150,000,000) were assessed using Tracer (Rambaut *et al.*, 2018). Tree files of independent runs were combined using LogCombiner (provided with BEAST2), summarized

with TreeAnnotator (provided with BEAST2), and visualized with FigTree (https://github.com/rambaut/figtree/releases).

Genome annotation

We built custom repeat libraries to identify repetitive elements across the genome. Discovery of TEs based on structural features was achieved using EDTA v1.8.3 (Ou *et al.*, 2019), which runs LTRharvest and LTR_retriever to identify LTR-RTs, TIR-Learner for TIR transposons and Helitronscanner for helitrons, and produces a filtered non-redundant TE library. We identified other repetitive elements using RECON (version 1.08) (Bao & Eddy, 2002) and RepeatScout (version 1.06) (Price *et al.*, 2005) as implemented within the RepeatModeler package (version 2.0) (Flynn *et al.*, 2020), producing a curated *B. laevigata* custom TE library.

Automated annotation of the reference genome utilized RepeatMasker (Smit *et al.*, 2015) with the *B. laevigata* custom TE library, protein datasets from Swissprot Viridiplantae and RNA-seq data generated from *B. laevigata* using leaf transcriptomes in response to environmental treatments (see "gene expression in response to environmental changes") as well as flash frozen tissue from roots, young leaves (2-7 days following leaf blade emergence), senescent leaves (2-4 days after the appearance of yellow sectors), stems, apical meristem, floral buds (2-4 days prior to anthesis) and open flowers (1-3 days post-anthesis) collected on a sample grown under greenhouse conditions (European Nucleotide Archive accession: PRJEB48599). For each sample, 1 - 5 µg of total RNA isolated using the RNAeasy mini kit (Qiagen) was prepared using Illumina TruSeq Stranded RNA library and sequenced on one flow cell of an Illumina HiSeq3000 to generate 386 Mbp of 150 bp paired-end reads that were assembled using TRINITY (Grabherr *et al.*, 2011; Haas *et al.*, 2013), with default parameters and the strandedness parameter set to -SS_lib_type FR.

For *ab initio* gene prediction, SNAP v.2013-11-29 (Korf, 2004), Genemark v.4 (Bruna *et al.*, 2023) and Augustus v.3.3.3 (Stanke *et al.*, 2008) were used. Genemark and Augustus were trained with BRAKER v.2 (Bruna *et al.*, 2021) prior to running MAKER (Holt & Yandell, 2011). Mapped RNA-seq reads from seven tissues as well as leaf tissue under different environmental conditions and Swissprot protein sequences from Viridiplantae were used as homology-based evidence.

Later SNAP was trained using MAKER by first using the longest isoforms from the *B. laevigata* transcriptome and the pre-trained Genemark and Augustus models directly to infer gene predictions. MAKER was run iteratively three additional times using the transcriptomes as evidence and providing updated training files for each run. The *B. laevigata* repeat library was included to mask repetitive elements from annotation. The resulting set of predicted genes was annotated with Pfam domains (El-

Gebali *et al.*, 2019) using InterProScan version 5.4–47.0 (Jones *et al.*, 2014) and the models were filtered selecting them if they had an annotation edit distance < 0.5 and/or PFAM domain. The final annotation of the protein-coding genes, based on the predicted genes models, was made using GOMAP (Wimalanathan et al., 2021) and Trinotate (Griffith *et al.*, 2015). Signal peptides (secretion signals) and transmembrane domains were predicted using signalIP and tmhmm software tools, respectively. KEGG terms were annotated using eggNOG mapper and filtered to plant-specific terms with the KEGGREST R package.

Accordingly, it resulted in the high-quality annotation of 43,632 genes coding for at least 20 amino acids, with GO terms, Pfam domains, and an annotation edit distance below 0.5. Atleast 86.3% of complete BUSCO genes were identified in our annotation (C:86.3%[S:64.4%,D:21.9%],F:4.6%,M:9.1%,n:2326).

Gene expression in response to environmental changes

Leaf transcriptomes in response to environmental treatments (European Nucleotide Archive accession: PRJEB48469) were generated from clones of the sequenced individual subjected to control (22°C, 16/8h light/dark cycle and daily watering; 4 replicates), cold (24h at 4°C, 16/8h light/dark; 4 replicates), heat (3h gradual increase from 22-42°C and 6h at 42°C; 3 replicates), drought (control conditions following 11.5 days without watering; 3 replicates) and herbivory condition (30h of feeding by *Plutella xylostella* 3-5th instar larvae on leaves used for RNAseq; 3 replicates). Environmental treatments were designed to mimic data available for *A. thaliana* (19 RNAseq libraries) from the studies of Klepikova *et al*. (2016; i.e. cold and heat treatments), Dubois *et al*. (2017; drought treatment) and Nallu *et al*. (2018; herbivory treatment).

Transcriptome comparability out of the 17 libraries for *B. laevigata* was ensured through synchronous sampling of leaves. Strand-specific libraries were prepared from RNA extracts using the “TruSeq Stranded Total RNA with Ribo-Zero Plant”-kit (Illumina), including ribosomal RNA depletion and size selection of 300 bp fragments. The pool of 17 libraries was sequenced on two lanes of an S2 flow cell of the NovaSeq 6000 system to yield more than 1.1 billion raw 2 x 50 bp paired-end reads. Reads were processed before gene expression quantification using RSEM (Li & Dewey, 2011) and identification of differentially expressed genes (DEGs) presenting a log2-fold change of ≥1 at a Benjamini-Hochberg false discovery rate threshold of < 0.001, using edgeR (Robinson *et al.*, 2009).

Genes expressed at < 1 transcript per million were considered “unexpressed”, whereas genes expressed ≥ 1 TPM were considered “expressed”.

Dynamics of transposable elements (TEs)

We used parseRM.pl (https://github.com/4ureliek/Parsing-RepeatMasker-Outputs/blob/master/parseRM.pl) to summarize the Repeatmasker annotation of TEs in *B. laevigata* and calculate the percentage of divergence of each copy to the consensus following Maumus & Quesneville (2014). In case of overlaps (when a position could be aligned to more than one consensus sequence), the genomic abundance of each TE subclass was corrected by choosing the smallest percentage divergence for that position. We produced a landscape plot of the genome composition, where the TE-subclass composition is presented in 1% divergence windows (compared to each TE copy's respective consensus library sequence) (Kapusta *et al.*, 2017), where low-divergence sequences indicate recent insertions and higher divergence sequences suggest older insertions. We dated the divergence of each annotated TE to its consensus using the synonymous substitution rate of 8.22×10^{-9} substitutions/synonymous site/year for Brassicaceae species (Kagale *et al.*, 2014).

Synteny scans of duplicated chromosome segments

Duplicated copies of genes following the last WGD event were identified through analysis of gene collinearity within the Buckler Mustard genome and between *B. laevigata* and ancestral genomic blocks of Brassicaeae extracted from *A. thaliana* using the "SynMap" algorithm within the CoGe package for comparative genomics (https://genomevolution.org/CoGe/GEvo.pl) (Lyons *et al.*, 2008). First, genes of *B. laevigata* sharing syntenic orthologs with *A. thaliana* were determined using DAGchainer (Haas *et al.*, 2004) and quota align algorithms (Tang *et al.*, 2011). Duplicates within the Buckler Mustard genome were identified following an all-versus-all BLASTp of the *B. laevigata* gene set to itself. The following parameter settings were used: ten collinear genes to seed a syntenic block; and a maximum of 20 nonsyntenic genes between syntenic genes to interrupt genomic blocks as previously described (Woodhouse *et al.*, 2011; Tang *et al.*, 2011).

Signals of selection among retained genes

Synonymous substitutions rates (Ks) and non-synonymous substitutions rates (Ka) as compared to *A. thaliana* orthologs were determined using CoDeML of the PAML package (Yang, 2007) as implemented in SynMap. Selection regimes were defined according to the range of Ka/Ks values and its standard deviation (SD = 0.2133). Genes were considered to be under purifying selection when Ka/Ks ≤ 1-SD (i.e. ≤ 0.787), neutral when 1-SD < Ka/Ks ≤ 1+SD (i.e. between 0.787 and 1.213) and under positive selection when Ka/Ks > 1+SD (i.e. > 1.213).

Detection and dating of WGD events

The simultaneous duplication of all genes during WGD results in an approximately Gaussian distribution of Ks values among pairs of duplicates (Blanc & Wolfe, 2004) and was here detected using mixture models that fits normal distributions to multimodal data (Barker *et al.*, 2009). Following removal of pairs of duplicates with Ks>2 or Ks< 0.01 that proved less reliable for inference of WGDs (Vanneste *et al.*, 2013), normal peaks in the distribution of Ks were inferred using the R package mixtools (Benaglia *et al.*, 2009). The most likely number of normal distributions in the observed distribution of Ks among pairs of duplicates was tested by a parametric bootstrap analysis (10 bootstraps) comparing the likelihood ratio of k versus k + 1 in between k = 1 and k = 5. Then, the iterative "expectation-maximization" algorithm (i.e., normalmixEM procedure) converged to the positions and standard deviations of two normal distributions.

Converting Ks time equivalents into absolute time using the α-WGD event (mean Ks = 0.96) as a calibration point offered rough estimates of the time of duplication events. Following Hohmann et al. (2015), 32.42 million years was set as the minimum age of the α-WGD event (Lysak & Koch, 2011).

Identification of the most-fractionated (MF) and least-fractionated (LF) subgenomes

Multiple chromosomal segments in *B. laevigata* that are orthologous to the same ancestral genomic blocks extracted from *A. thaliana* were compared to one another. All non-overlapping windows of syntenic genes in *B. laevigata* that matched to the same segment in *A. thaliana* were partitioned into two sub-genomes. Corresponding segments of the two subgenomes in *B. laevigata* were classified as "Least Fractionated" (LF) and "Most Fractionated" (MF) according to their phylogenetic placement along with cds from *A. thaliana* and *M. pygmaea*, and an inhouse assembled transcriptome of *Heldreichia bupleurifolia* – the sister genus to *Biscutella*. We assigned orthologous genes of *M. pygmaea* and *H. bupleurifolia* to *A. thaliana* using reciprocal best blast hit and then used the orthologous gene along with the duplicated syntenic gene copies of *B. laevigata* to make codon aware alignments using pal2nal. Inferring phylogenetic trees rooted on *A. thaliana* genes using RAxML, we followed Guo et al. (2021) and labelled the duplicated genes being sister to *Heldreichia* as subgenome A, whereas the other duplicate was labeled as subgenome B. High node support gene trees (>70% bootstrap value) were used to assign the windows corresponding to the LF and the MF subgenomes.

Duplicated windows from each subgenome were further partitioned into "low-bias" regions presenting quasi-unbiased fractionation (i.e. with similar number of retained syntenic genes in the MF

and LF subgenomes) and “high-bias” regions undergoing heavily biased fractionation (i.e. significantly different number of retained syntenic genes between the MF and LF subgenomes) based on chi-squared test (non-significant differences in proportion of retained genes in MF and LF (p-value > 0.05) classified as “low-bias”, and significant difference classified as “high-bias”). Differences in the number of retained TEs, the proportion of retained TEs to retained syntenic genes, and the proportion of differentially expressed duplicated genes per retained syntenic genes were compared among these different sets.

References

Bao Z, Eddy SR. 2002. Automated *de novo* identification of repeat sequence families in sequenced genomes. Genome Research 12: 1269–1276.

Barker MS, Vogel H, Schranz ME. 2009. Paleopolyploidy in the Brassicales: Analyses of the cleome transcriptome elucidate the history of genome duplications in Arabidopsis and other Brassicales. Genome Biology and Evolution 1: 391–399.

Benaglia T, Chauveau D, Hunter DR, Young D. 2009. Mixtools: An R package for analyzing finite mixture models. Journal of Statistical Software 32: 1-29.

Blanc G, Wolfe KH. 2004. Functional divergence of duplicated genes formed by polyploidy during Arabidopsis evolution. The Plant cell 16: 1679–1691.

Bouckaert R, Vaughan TG, Barido-Sottani J, Duchêne S, Fourment M, Gavryushkina A, Heled J, Jones G, Kühnert D, De Maio N, et al. 2019. BEAST 2.5: An advanced software platform for Bayesian evolutionary analysis. PLOS Computational Biology 15: e1006650.

Bruna T, Lomsadze A, Borodovsky M. 2023. GeneMark-ETP: Automatic gene finding in eukaryotic genomes in consistence with extrinsic data. BioRxiv: https://doi.org/10.1101/2023.01.13.524024

Dogan M, Pouch M, Mandáková T, Hloušková P, Guo X, Winter P, Chumová Z, Van Niekerk A, Mummenhoff K, Al-Shehbaz IA, Mucina L and Lysak MA. 2021. Evolution of tandem repeats is mirroring post-polyploid cladogenesis in *Heliophila* (Brassicaceae). Frontiers in Plant Science 11: 607893.Doležel J, Greilhuber J, Suda J. 2007. Estimation of nuclear DNA content in plants using flow cytometry. Nature Protocols 2: 2233-2244.

Dubois M, Claeys H, van den Broeck L, Inzé D. 2017. Time of day determines Arabidopsis transcriptome and growth dynamics under mild drought. Plant Cell and Environment 40: 180–189.

El-Gebali S, Mistry J, Bateman A, Eddy SR, Luciani A, Potter SC, Qureshi M, Richardson LJ, Salazar GA, Smart A, et al. 2019. The Pfam protein families database in 2019. Nucleic Acids Research 47: D427–D432.

Flynn JM, Hubley R, Goubert C, Rosen J, Clark AG, Feschotte C, Smit AF. 2020. RepeatModeler2 for automated genomic discovery of transposable element families. Proceedings of the National Academy of Sciences 117: 9451–9457.

Geiser C, Mandáková T, Arrigo N, Lysak MA, Parisod C. 2016. Repeated whole-genome duplication, karyotype reshuffling, and biased retention of stress-responding genes in Buckler Mustard. Plant Cell 28: 17–27.

Grabherr MG, Haas BJ, Yassour M, Levin JZ, Thompson DA, Amit I, Adiconis X, Fan L, Raychowdhury R, Zeng Q, et al. 2011. Full-length transcriptome assembly from RNA-Seq data without a reference genome. Nature Biotechnology 29: 644–652.

Griffith M, Walker JR, Spies NC, Ainscough BJ, Griffith OL. 2015. Informatics for RNA Sequencing: A Web Resource for Analysis on the Cloud. PLOS Computational Biology 11: 1–20.

Guo X, Mandáková T, Trachtová K, Özüdoğru B, Liu J, Lysak MA. 2021. Linked by ancestral bonds: multiple whole-genome duplications and reticulate evolution in a Brassicaceae tribe. Molecular Biology and Evolution 38: 1695–1714.

Haas BJ, Delcher AL, Wortman JR, Salzberg SL. 2004. DAGchainer: a tool for mining segmental genome duplications and synteny. Bioinformatics 20: 3643–3646.

Haas BJ, Papanicolaou A, Yassour M, Grabherr M, Blood PD, Bowden J, Couger MB, Eccles D, Li B, Lieber M, et al. 2013. *De novo* transcript sequence reconstruction from RNA-seq using the Trinity platform for reference generation and analysis. Nature Protocols 8: 1494–1512.

Hohmann N, Wolf EM, Lysak MA, Koch MA. 2015. A time-calibrated road map of Brassicaceae species radiation and evolutionary history. The Plant Cell 27: 2770-2784.

Holt C, Yandell M. 2011. MAKER2: an annotation pipeline and genome-database management tool for second-generation genome projects. BMC Bioinformatics 12: 491.

Huang C-H, Sun R, Hu Y, Zeng L, Zhang N, Cai L, Zhang Q, Koch MA, Al-Shehbaz I, Edger PP, et al. 2016. Resolution of Brassicaceae phylogeny using nuclear genes uncovers nested radiations and supports convergent morphological evolution. Molecular Biology and Evolution 33: 394–412.

Jones P, Binns D, Chang H-Y, Fraser M, Li W, McAnulla C, McWilliam H, Maslen J, Mitchell A, Nuka G, et al. 2014. InterProScan 5: genome-scale protein function classification. Bioinformatics 30: 1236–1240.

Kagale S, Robinson SJ, Nixon J, Xiao R, Huebert T, Condie J, Kessler D, Clarke WE, Edger PP, Links MG, et al. 2014. Polyploid evolution of the Brassicaceae during the Cenozoic era. The Plant Cell 26: 2777–2791.

Kajitani R, Yoshimura D, Okuno M, Minakuchi Y, Kagoshima H, Fujiyama A, Kubokawa K, Kohara Y, Toyoda A, Itoh T. 2019. Platanus-allee is a *de novo* haplotype assembler enabling a comprehensive access to divergent heterozygous regions. Nature Communications 10: 1702.

Kapusta A, Suh A, Feschotte C. 2017. Dynamics of genome size evolution in birds and mammals. Proceedings of the National Academy of Sciences 114: E1460-E1469.

Katoh K, Standley DM. 2013. MAFFT multiple sequence alignment software version 7: Improvements in performance and usability. Molecular Biology and Evolution 30: 772–780.

Klepikova A V, Kasianov AS, Gerasimov ES, Logacheva MD, Penin AA. 2016. A high resolution map of the Arabidopsis thaliana developmental transcriptome based on RNA-seq profiling. Plant Journal 88: 1058–1070.

Lefort V, Longueville J-E, Gascuel O. 2017. SMS: Smart model selection in PhyML. Molecular Biology and Evolution 34: 2422–2424.

Li B, Dewey CN. 2011. RSEM: accurate transcript quantification from RNA-Seq data with or without a reference genome. BMC Bioinformatics 12: 323.

Lieberman-Aiden E, van Berkum NL, Williams L, Imakaev M, Ragoczy T, Telling A, Amit I, Lajoie BR, Sabo PJ, Dorschner MO, et al. 2009. Comprehensive mapping of long-range interactions reveals folding principles of the human genome. Science 326: 289–293.

Lyons E, Pedersen B, Kane J, Freeling M. 2008. The value of nonmodel genomes and an example using SynMap within CoGe to dissect the hexaploidy that predates the Rosids. Tropical Plant Biology 1: 181–190.

Lysak MA, Koch MA. 2011. Phylogeny, Genome, and Karyotype Evolution of Crucifers (Brassicaceae). In: Genetics and Genomics of the Brassicaceae. New York, NY: Springer New York, 1–31.

Lysak MA, Mandáková T, Schranz ME. 2016. Comparative paleogenomics of crucifers: ancestral genomic blocks revisited. Current Opinion in Plant Biology 30: 108–115.

Mandáková T, Pouch M, Brock JR, Al-Shehbaz IA, Lysak MA 2019. Origin and evolution of diploid and allopolyploid *Camelina* genomes was accompanied by chromosome shattering. Plant Cell 31: 2596–2612.

Manni M, Berkeley MR, Seppey M, Simão FA, Zdobnov EM. 2021. BUSCO Update: Novel and streamlined workflows along with broader and deeper phylogenetic coverage for scoring of eukaryotic, prokaryotic, and viral genomes. Molecular Biology and Evolution 38: 4647–4654.

Maumus F, Quesneville H. 2014. Ancestral repeats have shaped epigenome and genome composition for millions of years in *Arabidopsis thaliana*. Nature Communication 5: 4104.

Nallu S, Hill JA, Don K, Sahagun C, Zhang W, Meslin C, Snell-Rood E, Clark NL, Morehouse NI, Bergelson J, et al. 2018. The molecular genetic basis of herbivory between butterflies and their host plants. Nature Ecology & Evolution 2: 1418–1427.

Ou S, Su W, Liao Y, Chougule K, Agda JRA, Hellinga AJ, Lugo CSB, Elliott TA, Ware D, Peterson T, et al. 2019. Benchmarking transposable element annotation methods for creation of a streamlined, comprehensive pipeline. Genome Biology 20: 275.

Price AL, Jones NC, Pevzner PA. 2005. *De novo* identification of repeat families in large genomes. Bioinformatics 21: i351–i358.

Pryszcz LP, Gabaldón T. 2016. Redundans: an assembly pipeline for highly heterozygous genomes. Nucleic Acids Research 44: e113–e113.

Putnam NH, O'Connell BL, Stites JC, Rice BJ, Blanchette M, Calef R, Troll CJ, Fields A, Hartley PD, Sugnet CW, et al. 2016. Chromosome-scale shotgun assembly using an in vitro method for long-range linkage. Genome Research 26: 342–350.

Rambaut A, Drummond AJ, Xie D, Baele G, Suchard MA. 2018. Posterior summarization in Bayesian phylogenetics using Tracer 1.7. Systematic Biology 67: 901–904.

Robinson MD, McCarthy DJ, Smyth GK. 2009. edgeR: a Bioconductor package for differential expression analysis of digital gene expression data. Bioinformatics 26: 139–140.

Smit A, Hubley R, Green P. 2015. RepeatMasker Open-4.0. http://www.repeatmasker.org.

Stanke M, Diekhans M, Baertsch R, Haussler D. 2008. Using native and syntenically mapped cDNA alignments to improve de novo gene finding. Bioinformatics 24: 637–644.

Tang H, Lyons E, Pedersen B, Schnable JC, Paterson AH, Freeling M. 2011. Screening synteny blocks in pairwise genome comparisons through integer programming. BMC Bioinformatics 12: 102.

Temsch EM, Koutecký P, Urfus T, Šmarda P, Doležel J. 2022. Reference standards for flow cytometric estimation of absolute nuclear DNA content in plants. Cytometry A 101:710–724.

Vanneste K, van de Peer Y, Maere S. 2013. Inference of genome duplications from age distributions revisited. Molecular Biology and Evolution 30: 177–190.

Wimalanathan, K., Lawrence-Dill, C.J. 2021. Gene Ontology Meta Annotator for Plants (GOMAP). Plant Methods 17: 54.

Woodhouse MR, Tang H, Freeling M. 2011. Different gene families in *Arabidopsis thaliana* transposed in different epochs and at different frequencies throughout the rosids. The Plant Cell 23: 4241–4253.

Xu M, Guo L, Gu S, Wang O, Zhang R, Peters BA, Fan G, Liu X, Xu X, Deng L, et al. 2020. TGS-GapCloser: A fast and accurate gap closer for large genomes with low coverage of error-prone long reads. GigaScience 9: 1-11.

Yang Z. 2007. PAML 4: Phylogenetic analysis by maximum likelihood. Molecular Biology and Evolution 24: 1586–1591.

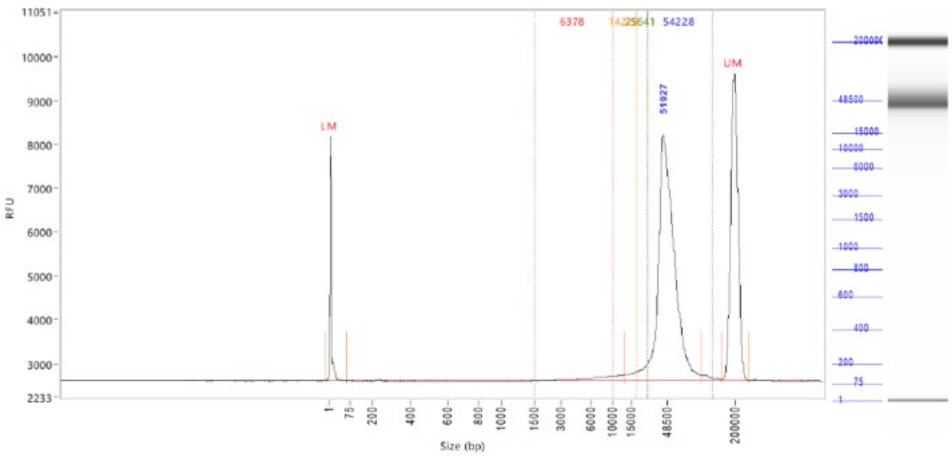

Figure S1.a. Biscutella laevigata austriaca HMW genomic DNA preparation. QC of gDNA quality by Fragment Analyzer resulted in ~90% gDNA > 30Kb

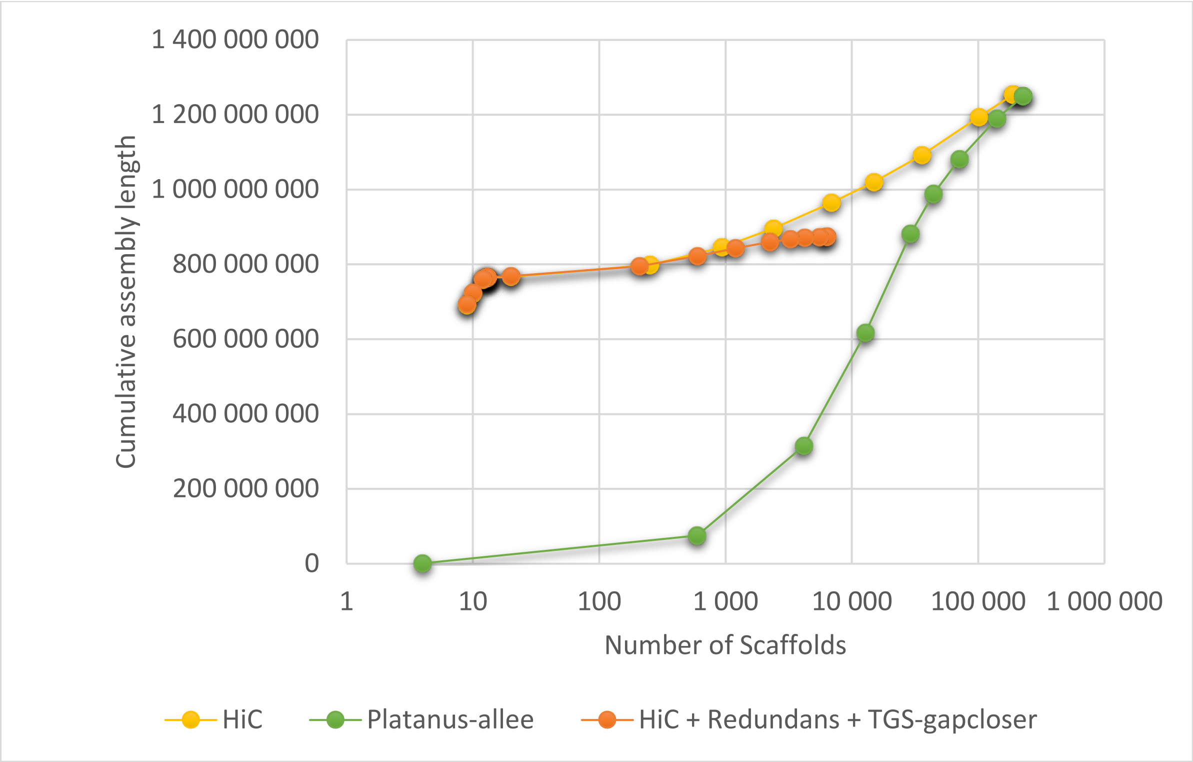

Figure S1.b. Three steps of genome assembly and improvements in cumulative assembly lengths. 1) Platanus-allee assembly resolved haplotigs with Illumina paired end reads, linked reads, and PacBio long reads. 2) HiC based scaffolding of the initial assembly led to improvement of assembly length with lower number of scaffolds. 3) Redundans and TGS-gapcloser step after HiC scaffolding removed remaining unassembled haplotigs and brought the assembly size closer to expectation.

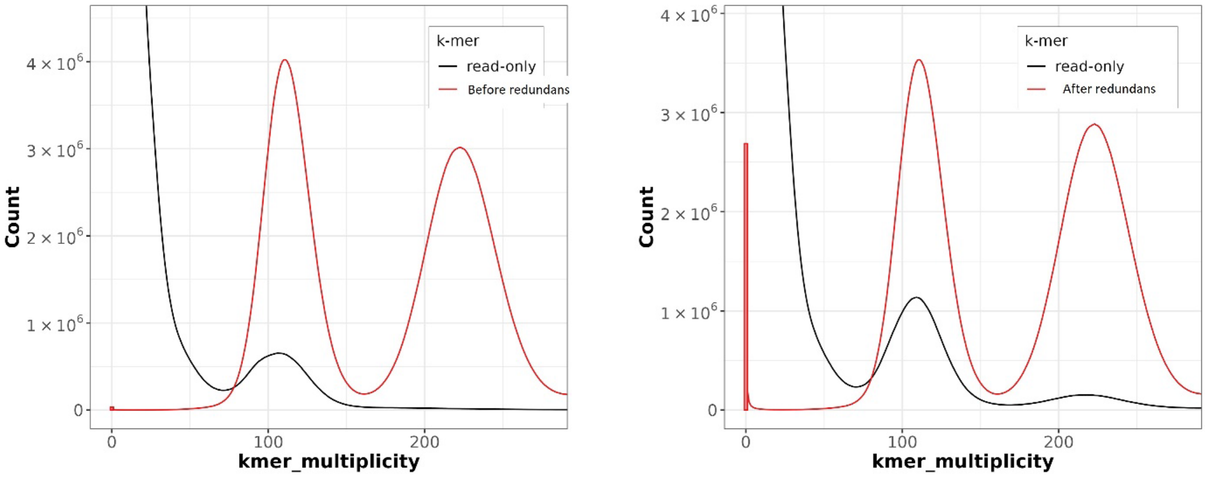

Figure S1.c. Mercury assembly evaluation of the genome assembly before and after Redundans based deduplication. Represents a reduction in heterozygous kmers compared to the assembly before Redundans.

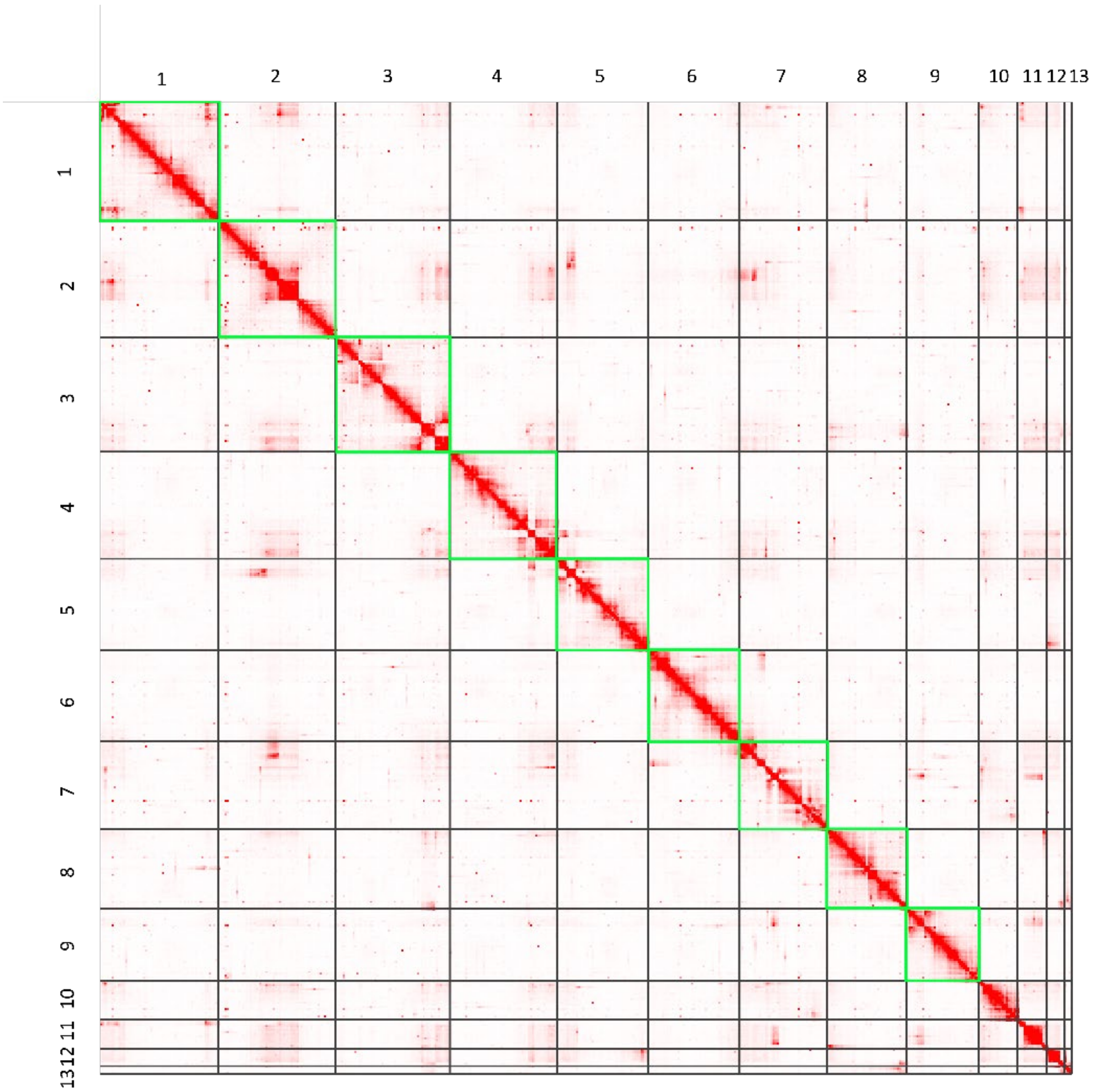

Figure S2.a. Hi-C contact map of the 13 assembled chromosome-length scaffolds submitted along with the manuscript (V1). Major 9 chromosome-length scaffolds were shown in green rectangle, corresponding to the 9 chromosomes in *B. laevigata* genome.

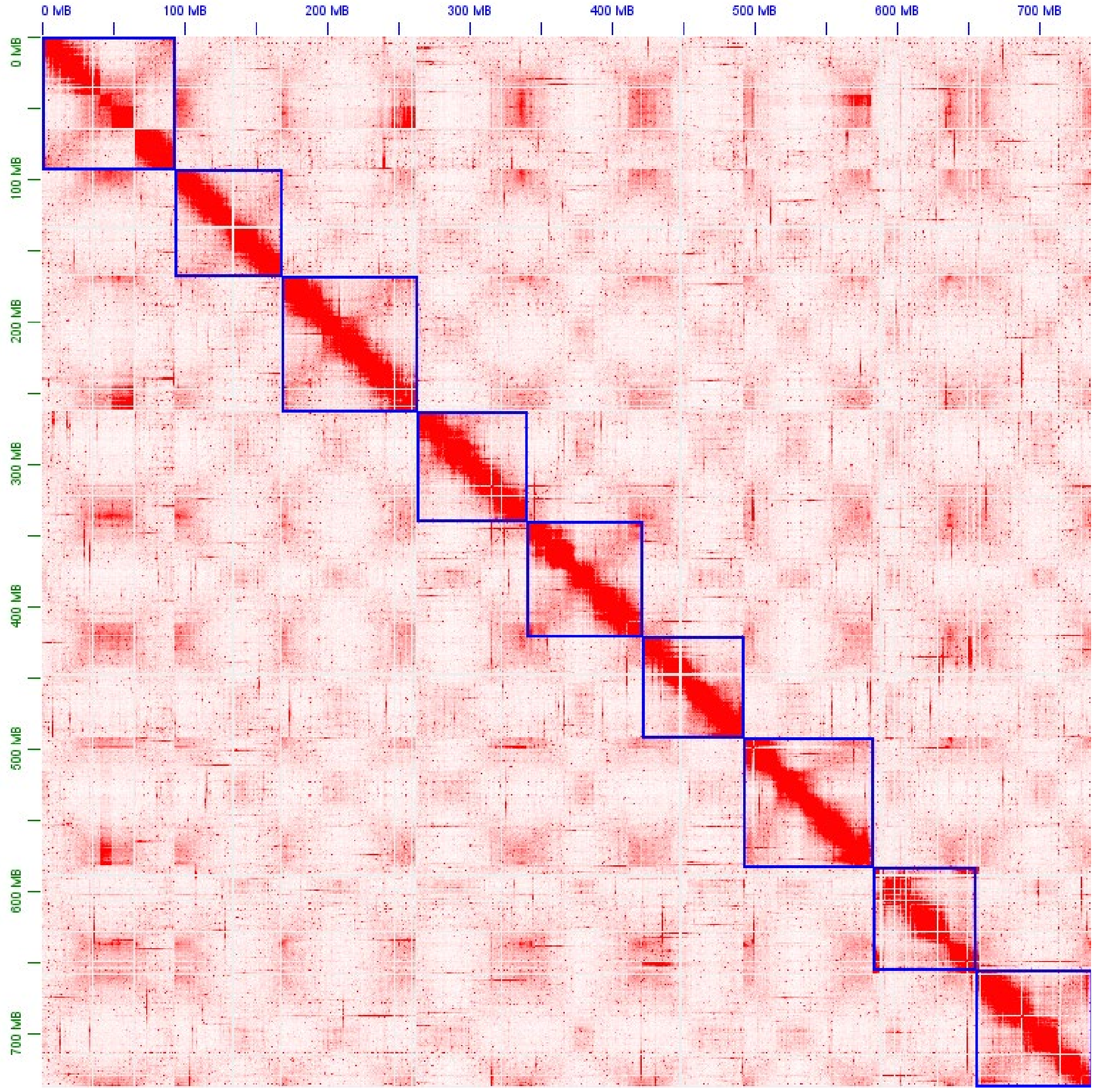

Figure S2.b. Hi-C contact map of the 9 assembled chromosome-length scaffolds, submitted as supplementary dataset (V2). Major 9 chromosome-length scaffolds were shown in blue rectangle, corresponding to the 9 chromosomes in *B. laevigata* genome.

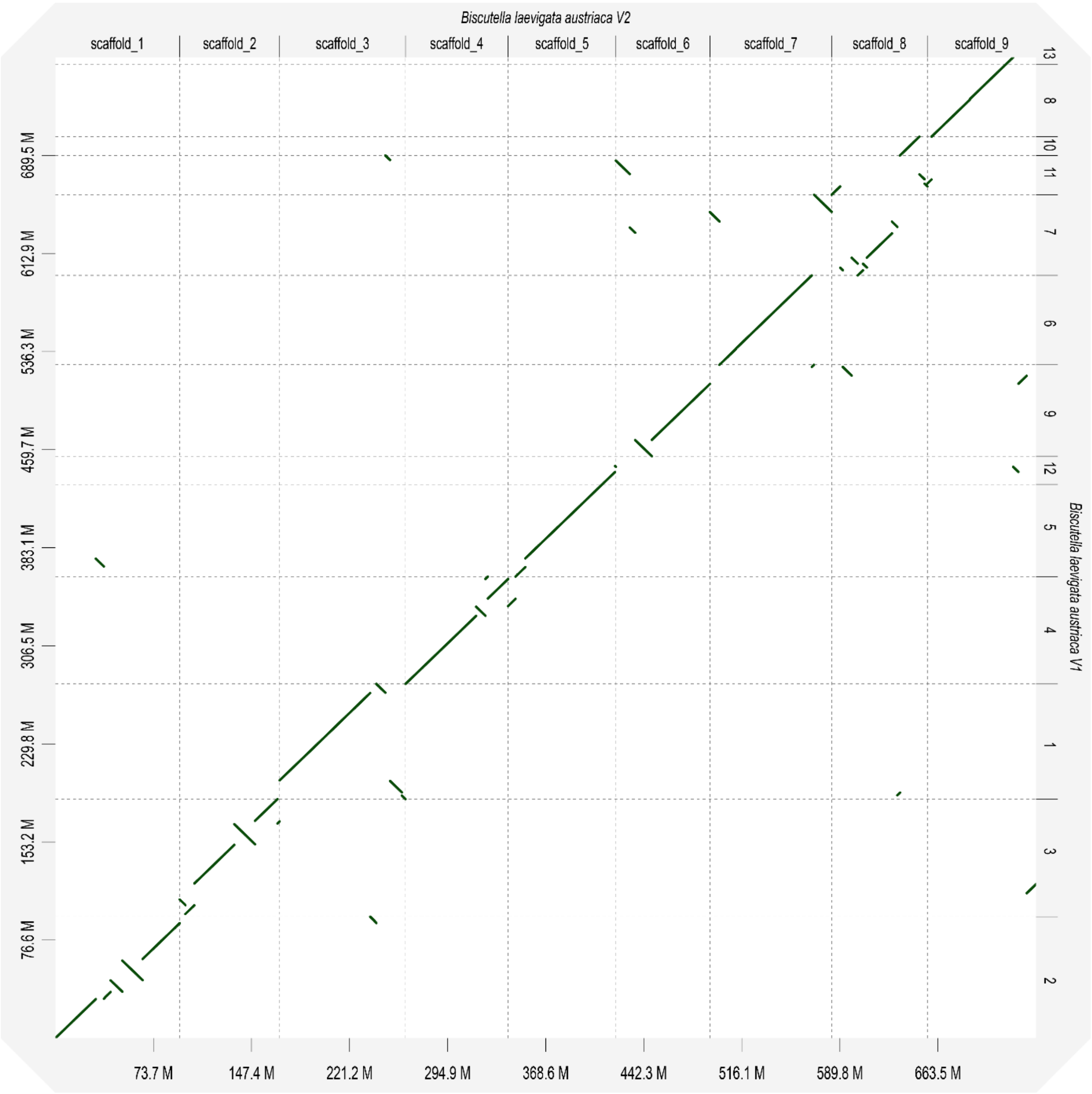

Figure S2.c. Dotplot between V1 and V2. An average of 7 Mb segments were rearranged between V1 and V2.

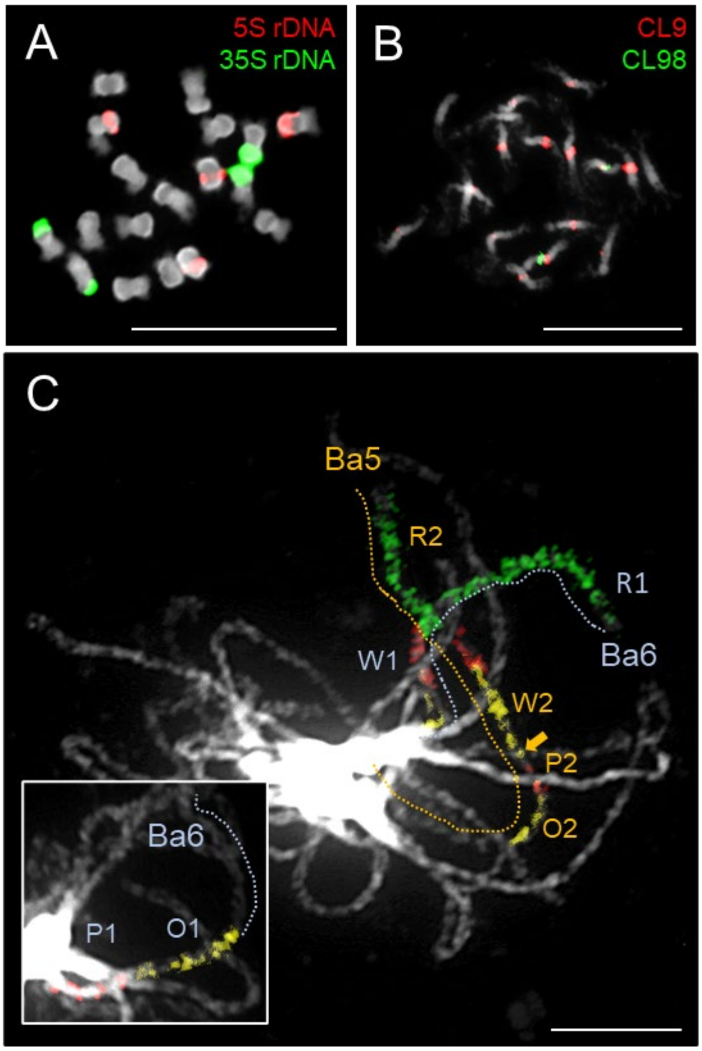

Figure S3: Chromosome analysis of *Biscutella laevigata* subsp. *austriaca*. (A) *In situ* localization of 5S and 35S rDNA loci on mitotic chromosomes (2n = 18). (B) *In situ* localization of the 213-bp (CL9) and 468-bp (CL98) centromeric tandem repeats on mitotic chromosomes. (C) An example of comparative chromosome painting. Chromosomes Ba5 and Ba6 in meiosis (pachytene) were painted using *A. thaliana* BAC contigs representing ancestral genomic blocks O, P, W, and R. The two genomic copies are discerned as #1 and #2. The arrow indicates the putative location of an inactive paleocentromere between blocks P2 and W2. Scale bars, 10 µm.

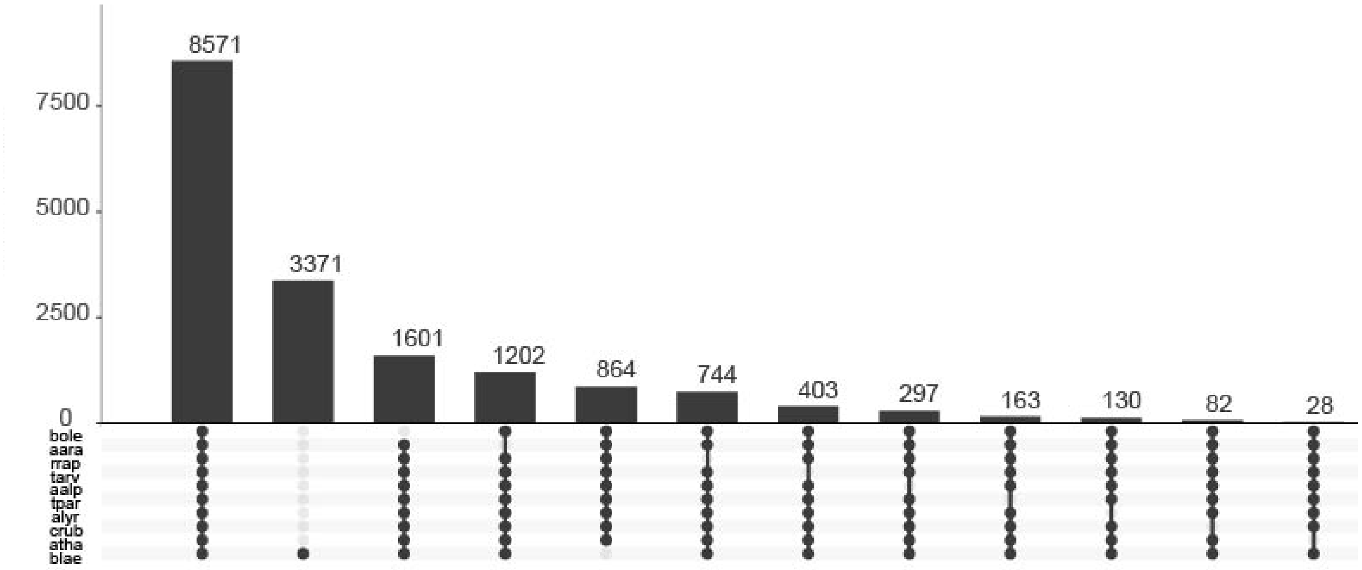

Figure S4: Upsetplot of Orthofinder-based othogroups in *Biscutella laevigata* shared by other Brassicaceae species. Orthologs shared between *Biscutella laevigata* (blae) and all or all-but-one other species in Brassicaceae studied here (*Arabidopsis thaliana*, atha; *Capsella rubella*, crub; *Arabidospis lyrata*, alyr; *Thellungiella parvula*, tpar; *Arabis alpina*, aalp; *Thlaspi arvensis*, tarv; *Raphanus raphanistrum*, rrap; *Aethionema arabicum*, aara; *Brassica oleracea*, bole) made the majority of orthogroups. However, *Biscutella* also presented several species-specific orthogroups.

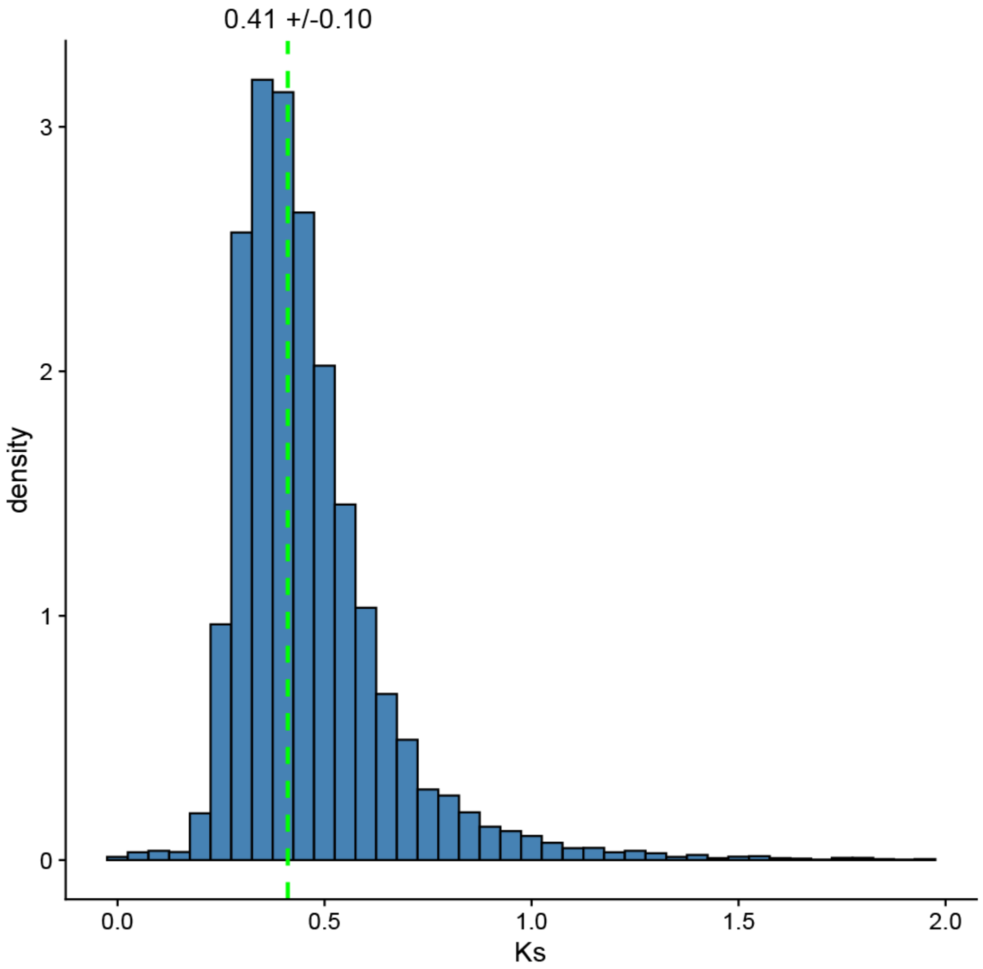

Figure S5. The Ks distribution of the orthologous pairs of genes between *B. laevigata* and *A. thaliana*. Mixture model estimated the mean peak at 0.41+/-0.10 which indicates the speciation event between these two species. This event is also indicated as a dashed green line in Fig 1c. This suggest that the identified WGD event in Fig. 1c occurred after the speciation event between *A. thaliana* and *B. laevigata*.

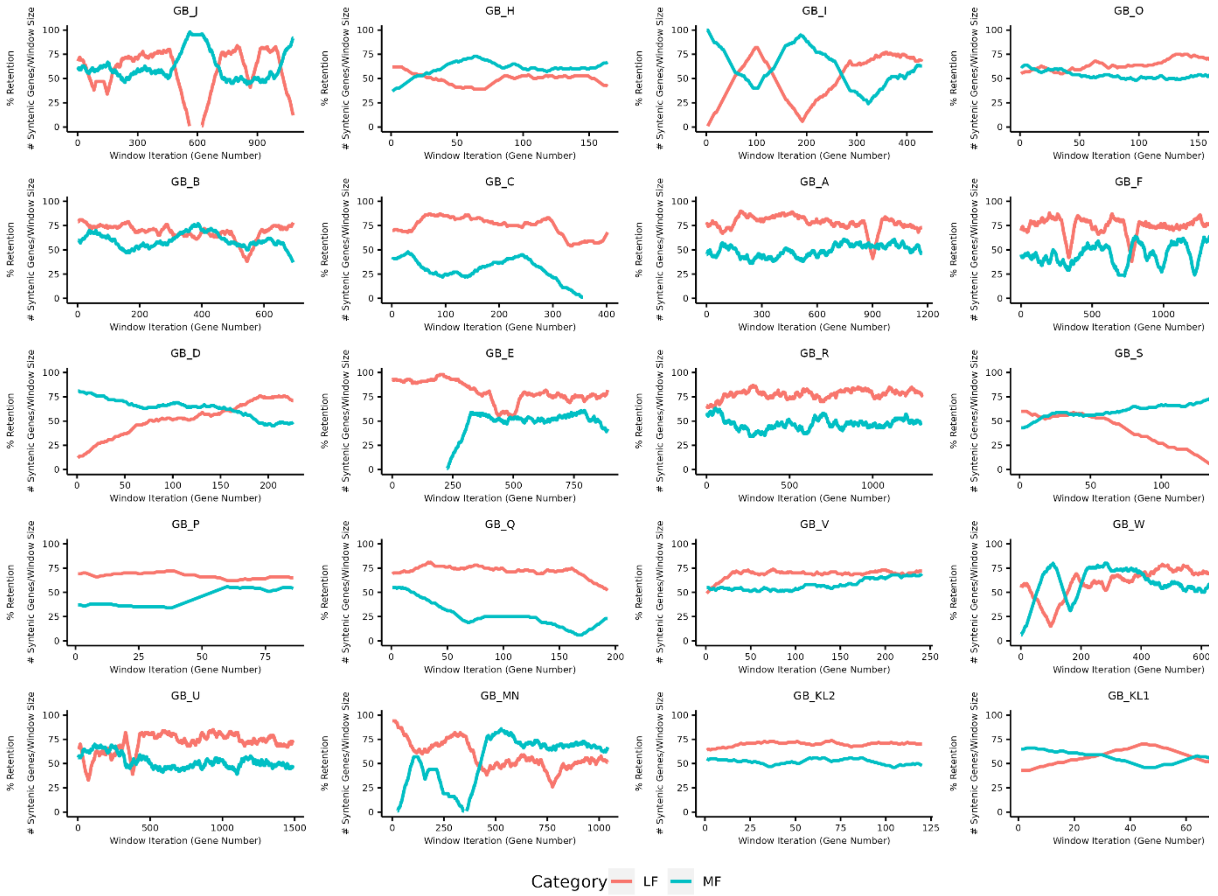

Figure S6.a. Identification of least fractionated (LF) and most fractionated (MF) subgenomes in *B. laevigata* based on *A. thaliana* genomic blocks (GB). The number of retained duplicates in the LF and MF subgenomes per 100 gene genomic windows across *A. thaliana* genomic blocks are represented in red and blue, respectively.

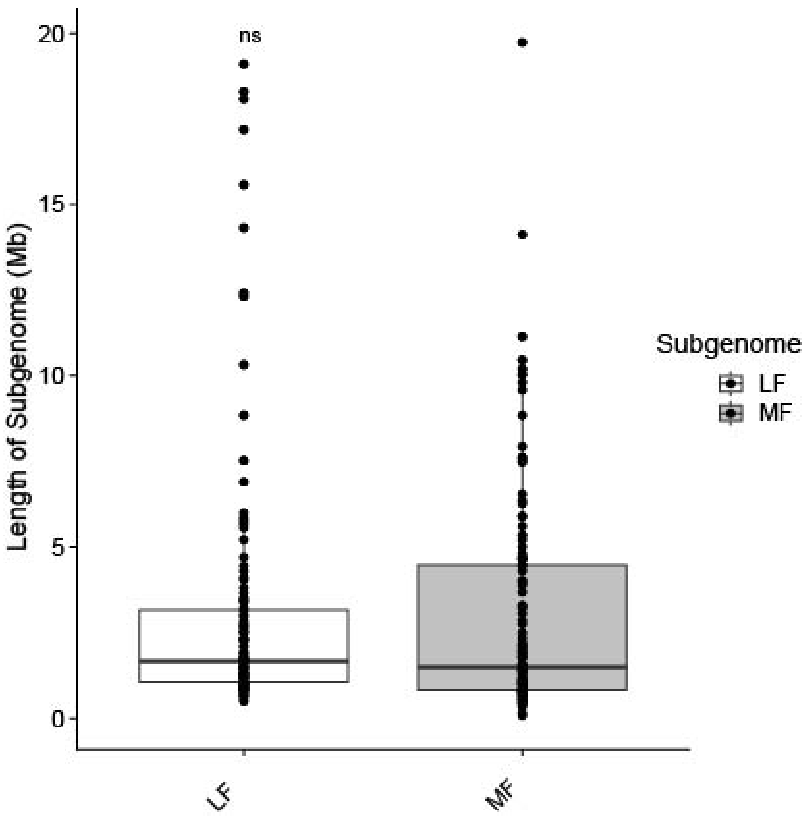

Figure S6.b. Size distribution of syntenic windows identified in the least fractionated (LF) and most fractionated (MF) subgenome of *B. laevigata* (each corresponding to 100 syntenic A. thaliana genes). The median length of the analysed widows is 1.58 Mb.

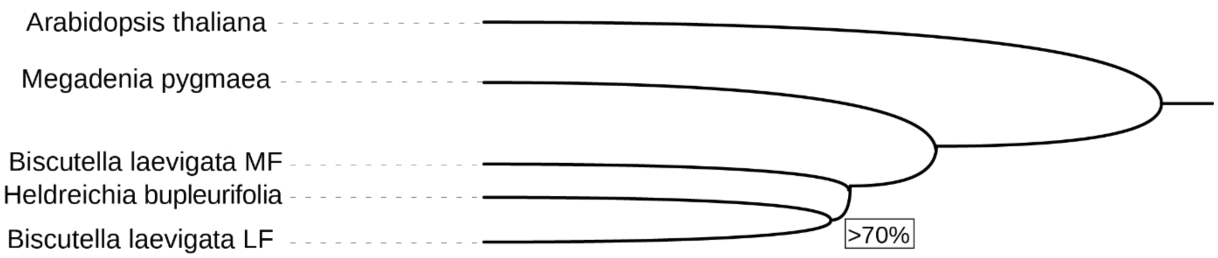

Figure S7. Identification of least fractionated (LF) and most fractionated (MF) subgenomes in *Biscutella laevigata* based on phylogenetic placement. The classification of duplicated genes was done according to the local topologies within individual gene trees. For each pair of genes in *Biscutella*, one of the duplicated genes was labeled as LF if it was sister to *Heldreichia* with a node support of > 70%, the other duplicate was labeled as MF.

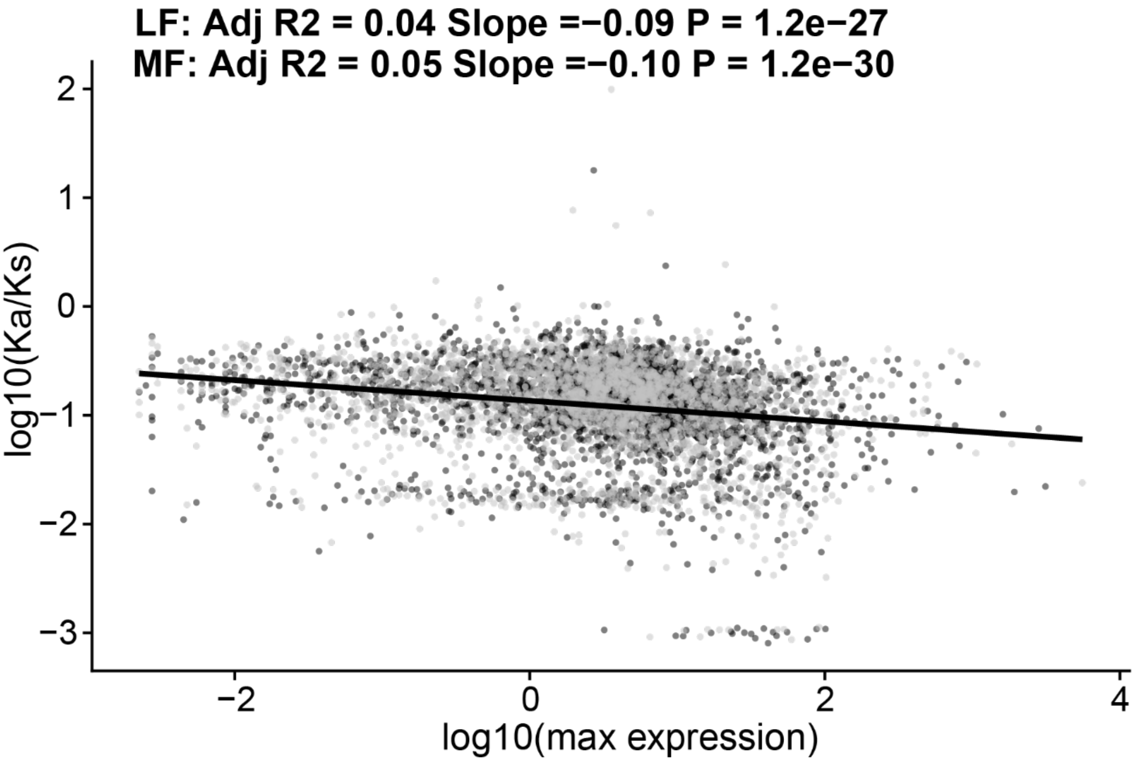

Figure. S8: Linear regression of signals of selection (Ka/Ks ratios) and maximum gene expression in *Biscutella laevigata*. A significant relationship with stronger signals of purifying selection (i.e. lower values of log10(Ka/Ks) in genes with higher expression for both least fractionated (LF) and most fractionated (MF) subgenomes is observed.

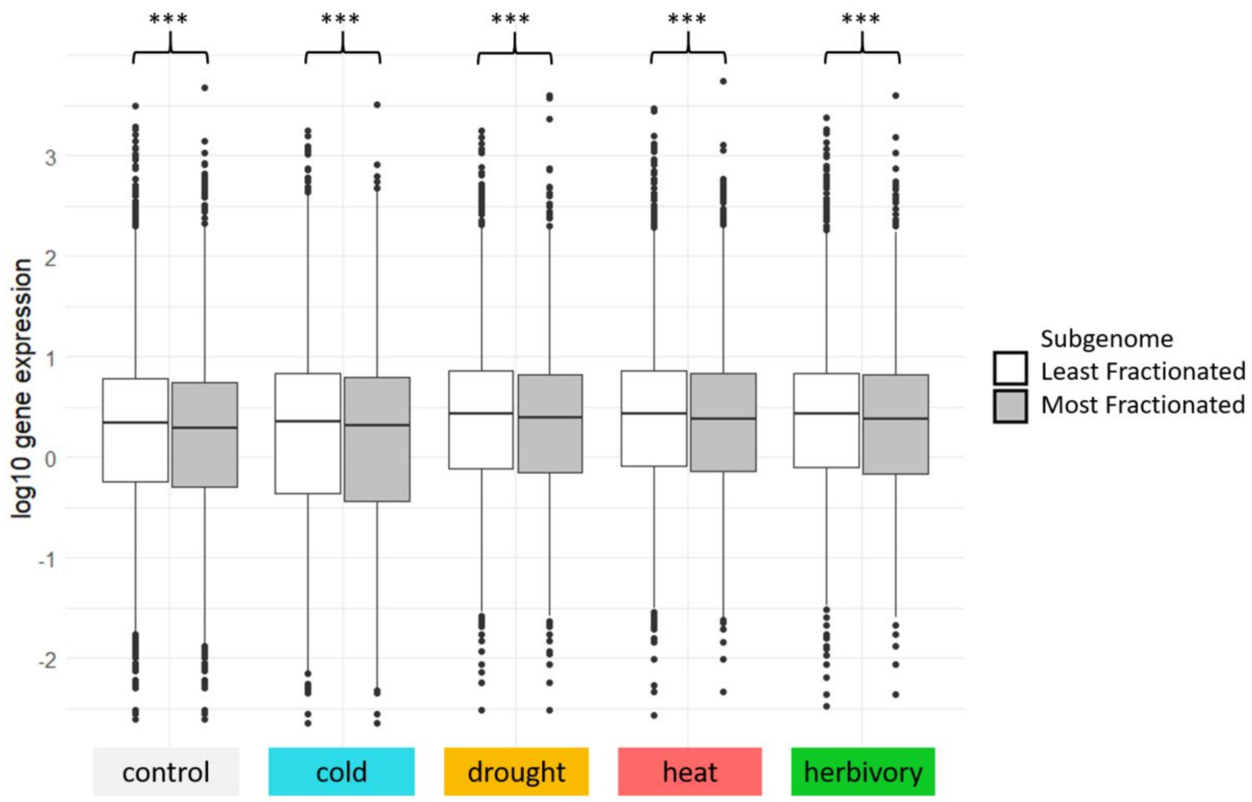

Figure S9. Expression of genes in the least fractionated and most fractionated subgenome of the mesopolyploid *Biscutella laevigata* in five different environmental treatments. Clones of the mesopolyploid *B. laevigata* individual from which we produced the *B. laevigata* genome assembly were subjected to control, cold, drought, heat and herbivory environments in Beringer *et al.* (2023). The average gene expression was significantly higher for genes in the LF subgenome compared with the MF subgenome, in all investigated environments (Wilcoxon test p-values < 0.001). N.B. Zero-expressed genes were removed from the plot for clarity, but not for the statistical analyses. Significance of Wilcoxon tests represented as p-value < 0.001 (***), < 0.01 (**), < 0.05 (*) and non-significant (ns).

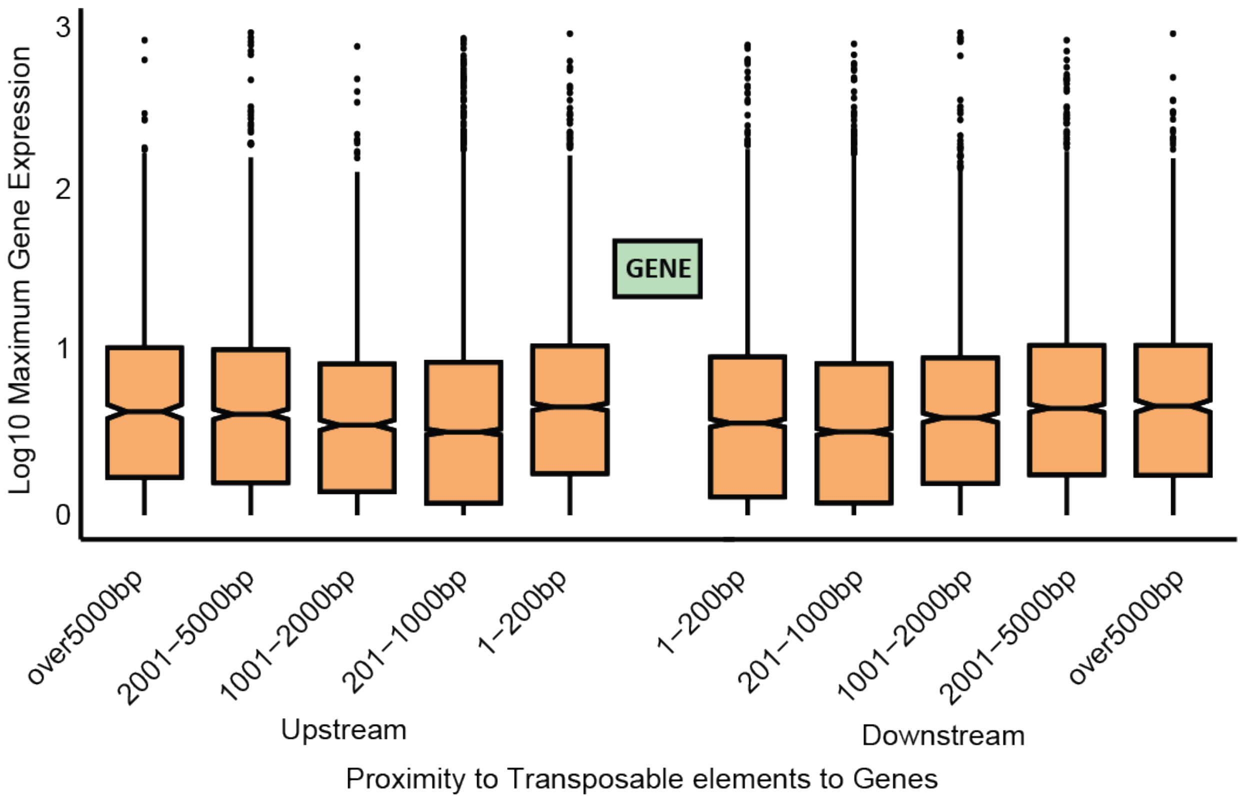

Figure S10: Gene expression of genes which vary in their proximity to transposable elements (TE) in the mesopolyploid *Biscutella laevigata* genome. Overall, lower expression levels were observed when TEs were between 200 and 2000 bp upstream or downstream of genes (see also Table S7). Gene expression levels when TEs were present more than 2000-5000 bp upstream or downstream were similar to expression levels observed when TEs were far from genes (i.e. > 5000bp).
